# Environmental selection constrains metabolic network architecture despite taxonomic turnover in anaerobic digestion communities

**DOI:** 10.1093/ismejo/wrag145

**Published:** 2026-06-08

**Authors:** Lisa Jourdain, Aaron Leininger, Alan R Pacheco, Wenyu Gu

**Affiliations:** Institute of Environmental Engineering, School of Architecture, Civil and Environmental Engineering, Swiss Federal Institute of Technology Lausanne, 1015 Lausanne, Vaud, Switzerland; Institute of Environmental Engineering, School of Architecture, Civil and Environmental Engineering, Swiss Federal Institute of Technology Lausanne, 1015 Lausanne, Vaud, Switzerland; Department of Environmental Microbiology, Swiss Federal Institute of Aquatic Science and Technology, 8600 Dübendorf, Zürich, Switzerland; Department of Fundamental Microbiology, University of Lausanne, 1015 Lausanne, Vaud, Switzerland; Institute of Environmental Engineering, School of Architecture, Civil and Environmental Engineering, Swiss Federal Institute of Technology Lausanne, 1015 Lausanne, Vaud, Switzerland

**Keywords:** microbial ecology, anaerobic digestion, microbial interactions, functional guilds, functional redundancy, metabolic networks

## Abstract

Microbial ecosystems often sustain stable metabolic functions despite pronounced taxonomic turnover, yet the mechanisms underlying such reproducible functional states remain poorly understood. Here, we investigated how physicochemical constraints shape functional convergence in anaerobic digestion (AD) communities using replicated serial enrichments seeded from four distinct inocula. Across three pH levels and six substrate regimes, replicate communities from different inocula consistently converged toward reproducible metabolite profiles, with pH emerging as the dominant organizing factor. Community composition became progressively environment-driven over time, and after 30 generations, pH explained the largest fraction of compositional variance (permutational multivariate analysis of variance *R*^2^ = 0.21, *P* = .001), followed by substrate. Genome-resolved metagenomics revealed that convergence was accompanied by strong pH-dependent structuring of redox-balancing and terminal electron-sink pathways, whereas upstream carbohydrate-entry pathways were conserved. Taxonomic convergence was incomplete and scale-dependent: the ability to correctly assign communities to their inoculum declined from 75% at the genus level to 53% at the phylum level, indicating increasing similarity across inocula at coarser taxonomic resolution despite persistent fine-scale variability. Despite this taxonomic flexibility, communities assembled under identical conditions consistently recruited similar sets of metabolic pathways organized into comparable network architectures. Functional redundancy analyses showed high redundancy and flexible taxonomic implementation for upstream fermentative processes, contrasted with lower redundancy and stronger convergence for terminal methanogenic functions. Together, these results demonstrate that reproducible metabolic function in ad emerges from environmentally constrained assembly of shared metabolic network architectures, rather than deterministic fixation of species composition, highlighting environmental control of metabolic organization as a central principle governing microbiome function.

## Graphical abstract

**Graphical Abstract:**
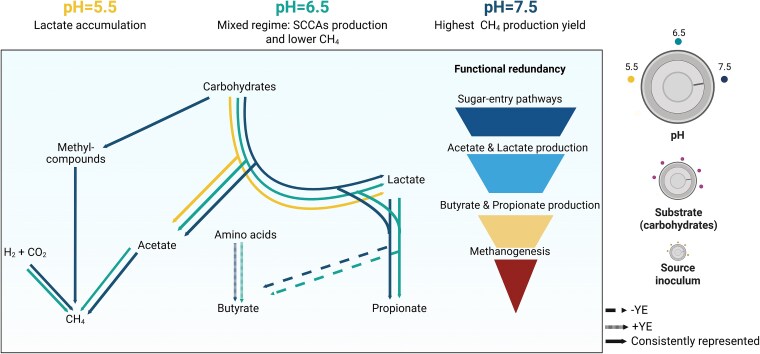

## Introduction

Microbial ecosystems sustain global biogeochemical fluxes by distributing metabolism across interdependent guilds. In soils, sediments, and gut environments alike, hundreds to thousands of taxa cooperate to drive organic matter degradation, intermediate turnover, and redox balance [1–5]. Despite substantial taxonomic variation, these communities often sustain stable metabolic outputs [6, 7] or recover comparable functional states following perturbation [8–10], even when phylogenetic composition diverges substantially [11, 12]. This recurrent decoupling between taxonomy and function, where “who is there” changes but “what they do” persists, has emerged as a defining feature of microbial ecosystems [7, 13–15].

However, the lack of a direct mapping between taxonomic composition and functional output complicates our ability to predict community behavior from species identity alone. If similar metabolic states arise from distinct assemblages of organisms, then reproducibility may reside at levels of organization beyond taxonomy. Evidence from natural microbiomes and enrichment experiments shows that functional profiles can converge independently of species identity, indicating that environmental selection acts primarily on functional organization rather than on the stabilization of specific taxa, thereby constraining the set of metabolic pathways and interaction structures available to phylogenetically distinct organisms [13, 16–20]. However, it remains unclear whether such functional convergence reflects stabilization of a shared underlying metabolic architecture or instead arises from multiple, alternative pathway-level organizations that yield equivalent functional outcomes.

Ecological theory frames community assembly as the outcome of deterministic filters—such as pH, substrate type, and redox state—interacting with stochastic processes including founder effects and demographic noise [21–26]. Deterministic filters constrain which metabolic strategies are thermodynamically and stoichiometrically feasible, whereas stochasticity influences which taxa occupy those strategies. Within a given functional state, multiple organisms can encode and perform equivalent metabolic functions [7, 17]. Functional guilds may be phylogenetically cohesive [19] or polyphyletic, comprising distantly related taxa sharing complementary traits [16, 20]. Closely related organisms can occupy distinct niches, whereas unrelated taxa may converge on similar roles [27–31]. However, these observations leave unresolved how functional convergence is mechanistically implemented: whether reproducible functional states arise through stabilization of shared pathway architectures, or instead through flexible reassembly of alternative pathway-level organizations that yield similar metabolic outputs.

Anaerobic digestion (ad) offers a powerful yet underexploited model system for dissecting these principles. Like soils and guts, ad microbiomes are trophically structured networks of fermenters, syntrophs, acetogens, and methanogens; exchanging metabolites and electrons through tightly coupled pathways [5, 32]. Large-scale surveys reveal extensive species diversity often comprising thousands of taxa per system, yet a consistent recurrence of higher-level functional guilds, suggesting that environmental context rather than taxonomy governs process performance [1]. Although inoculum history can shape early trajectories, differences tend to attenuate as communities reorganize under shared operating constraints [11, 33, 34].

ad communities are influenced by interacting physicochemical parameters, including pH [35], substrate inputs [36], ammonia levels [37], temperature [38], and hydraulic retention time (HRT) [39]. Thermodynamic constraints in ad do not act uniformly across metabolism, but instead tend to be most restrictive on syntrophic, redox-coupled steps. In particular, the oxidation of fermentation intermediates, such as short-chain carboxylates (SCCAs; e.g. lactate, propionate, butyrate, and formate) or ethanol, requires efficient removal of reducing equivalents via interspecies electron transfer, commonly mediated by hydrogen or formate [32, 40]. Consequently, variation in hydrogen partial pressure primarily modulates the feasibility of these syntrophic transformations and the routing of reducing equivalents at the community level [41]. As a result, environmental perturbations in ad primarily affect downstream redox balancing and terminal electron-accepting processes rather than carbon entry into metabolism. This energetic sensitivity implies that environmental filters can gate main metabolic regimes by constraining electron-disposal strategies and cross-feeding interactions [42]. ad therefore provides a tractable system to investigate how environmental conditions delimit alternative functional states, and whether convergence toward a given regime reflects conserved metabolic architectures or can arise through distinct pathway-level organizations.

Here, we investigate how these constraints shape functional convergence using time-resolved enrichments of ad consortia combining 16S rRNA gene sequencing, genome-resolved metagenomics, and metabolite profiling. Replicated microcosms seeded from four geographically and operationally distinct digesters were cultivated under defined pH (5.5, 6.5, 7.5), carbohydrate (glucose, cellobiose, xylose, and glucose-xylose mixtures), and nutrient regimes, and serially transferred over eight weekly batches. This factorial design reduces environmental complexity whereas preserving ad trophic structure, enabling us to disentangle the roles of environmental filtering and inoculum history in shaping community trajectories. We particularly examine community assembly through the lens of metabolic interactions and pathway organization among prokaryotic taxa. More specifically, we ask whether distinct inocula that converge toward similar metabolite profiles also converge toward shared metabolic pathway architectures, or arise through alternative pathway-level organizations. To address this, we conducted a pathway-resolved analysis of ad metabolism using a curated set of fermentative, syntrophic, and methanogenic routes. Rather than inferring function from gene presence alone, we examined the relative contribution of alternative metabolic pathways and their taxonomic carriers in the context of observed metabolite regimes. By quantifying functional redundancy within and between communities, we further assess the taxonomic scale at which functional predictability emerges during serial enrichment.

## Materials and methods

### Sampling locations

Four ad inocula were collected from geographically and functionally distinct digesters to capture variation in community composition and substrate history. Inoculum 1 was sourced from a biogas plant in Lower Saxony, Germany, processing maize silage and green rye (43°C; HRT 40–60 d), and Inoculum 2 from a mixed-feed biogas plant in the same region processing cattle manure and agricultural wastes (38°C; HRT 40–60 d). Inoculum 3 originated from a mesophilic wastewater treatment digester in Penthaz, Switzerland (35°C; HRT 20–30 d), and Inoculum 4 was obtained from the same facility nine months later. Samples were transported at 4°C, processed within one week, and archived at −80°C.

### Enrichment cultures and experimental conditions

**Figure 1:**
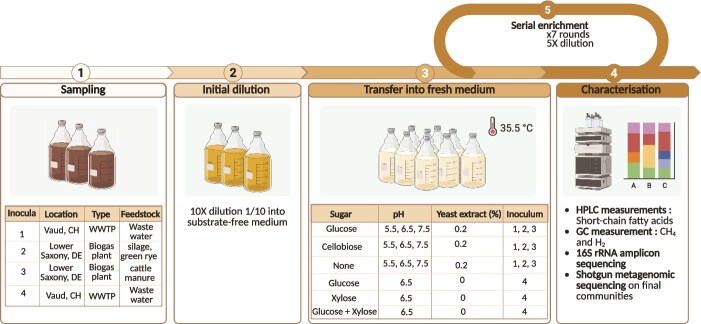
Design of enrichment experiments and multi-omics characterization. DE: Germany, CH: Switzerland, WWTP: wastewater treatment plant, H_2_: dihydrogen, CH_4_: methane.

Samples were diluted 1:10 into an organic carbon-free basal medium and incubated in biological triplicates under different substrate regimes: no carbon, glucose (15 mmol L^−1^), xylose (15 mmol L^−1^), glucose + xylose (7.5 mmol L^−1^ each), or cellobiose (7.5 mmol L^−1^) (Fig. 1). Substrates were selected to bypass hydrolysis and span distinct carbohydrate entry routes, with concentrations adjusted to ensure equal molar monomer inputs; yeast extract (0.2% w/v) was added where indicated. Prior to incubation, headspaces were purged with high-purity N₂ (99.999%) for 15 min to ensure strict anoxic conditions. Cultures were then incubated statically at 35.5°C with buffered pH, yielding 36 experimental conditions. Serial transfers were performed every 7 days by 1:5 dilution for eight batches, initiating at methane onset in batch 1 (except at pH 5.5), corresponding to ~30 generations based on OD_6_₀₀ estimates [43]. Biomass was harvested after each transfer and stored at −80°C. Detailed medium composition and calculations are provided in the Supplementary Information. Yeast extract was added where indicated to provide growth factors (vitamins, cofactors, and trace amino acids) required to sustain strict anaerobes over serial transfers [44–47]. Impacts of this addition on metabolic readouts are discussed in the Results section and in Supplementary.

pH was monitored at the end of each 7–10-day incubation period alongside OD_600_ and metabolite measurements. Despite buffering applied uniformly across all conditions (Supplementary Information), within-transfer pH shifts of 0.5–1.3 units were observed under highly fermentative conditions. These variations remained within their intended physiological regimes and did not cross critical functional transitions (e.g. methanogenic versus acidogenic states), as supported by the reproducible clustering of community composition and metabolite profiles across replicates and inocula. pH was restored to target values at each serial transfer by medium renewal. Accordingly, pH conditions are discussed throughout as controlled physiological regimes rather than strictly fixed-point values.

### Gas and volatile fatty acids measurements

Metabolite production was monitored via gas chromatography (Agilent 7890B; B-FATWAX Ultra Inert column; SRI Multigas 5 TCD-FID/Methanizer, USA) and high-performance liquid chromatography (Agilent 1260 Infinity II; Hi-Plex H column, USA) (Supplementary Information). pH (Metrohm, CH) and optical density (OD_600,_ Ultrospec, Biochrom, UK) were measured following standard procedures. Metabolite measurements are provided in Supplementary Tables S14 and S15.

### Deoxyribonucleic acid extraction

Cell pellets were thawed on ice, and DNA was extracted under sterile conditions using the DNeasy PowerSoil Pro Kit on a QIAcube automated platform (Qiagen, NL). The manufacturer’s protocol was followed with an additional lysis step (55°C, 5 min) and bead beating (2 × 20 s at 5000 rpm; Precellys, FR). Positive (ZymoBIOMICS Microbial Community Standard, USA) and negative extraction controls were included throughout. DNA concentrations were quantified using a Qubit fluorometer (Thermo Fisher Scientific, USA), and extracts were stored at −80°C until further processing. No enrichment of known low-biomass or kit contaminants was detected (Supplementary Fig. S1).

### 16 s ribosomal ribonucleic acid gene sequencing

PCR-free 16S rRNA gene library preparation and purification were performed as previously described [48]. Libraries were sequenced at the University of Lausanne Genomic Technologies Facility (UNIL-GTF) on an Aviti System (Element Biosciences, USA) using the Cloudbreak FS Standard Output 600-cycle kit, in paired-end 2 × 300 bp mode. Base calling and demultiplexing were carried out using bases2fastq (v2.0.0.1379264253). Taxonomic assignment was performed against the SILVA v138.2 database.

### Metagenomic survey and analysis

DNA extracts were sequenced at BGI Genomics (CN) using short-insert libraries on a DNBSEQ System (MGI tech, CH) (paired-end 150 bp). Sequencing yielded 38–40 million read pairs per sample (Q30 ≈ 94%–96%). Reads were processed using the nf-core/mag workflow [49], including quality filtering with fastp [50] and removal of phiX reads using Bowtie2 [51]. Assemblies were performed using MEGAHIT [52]. Metagenome-assembled genomes (MAGs) were recovered using MetaBAT2 [53] and MaxBin2 [54], refined and dereplicated with DAS Tool [55], and quality-assessed using CheckM2 [56]. MAGs with completeness ≥50% and contamination ≤10% were retained. Taxonomic classification was performed with GTDB-Tk [57] against the GTDB r226 database. Gene prediction and annotation were conducted using Prodigal [58], Prokka [59], and DRAM [60]. MAG abundances were inferred from contig-level sequencing depth files generated during assembly. For each sample, contig depths were mapped to their corresponding assigned bins using per-sample contig-to-bin tables, and genome-level coverage was computed as the length-weighted mean depth across all binned contigs, followed by within-sample normalization to derive relative abundance profiles. An overview of sample metadata, MAG quality metrics, taxonomic assignments, and relative abundances is provided in Supplementary Tables S1, S2, S3, and S4.

### Assembly-process inference and community-convergence analysis

Community assembly processes were quantified using a null-model framework [22], partitioning community turnover into deterministic and stochastic components. Reproducibility and convergence of community trajectories were assessed using dissimilarity-overlap analysis (DOA) [20]. Full methodological details, null-model implementations, and statistical thresholds are provided in the Supplementary Information.

### Definition of metabolic modules and functional redundancy calculation

Pathway presence thresholds were calibrated independently for each pathway category through sensitivity analyses (Supplementary Fig. S16). For butyrate production, a completeness threshold of ≥0.80 was applied to four routes: acetyl-CoA condensation (P1), 4-aminobutyrate/4-hydroxybutyrate (P2), glutarate (P3), and lysine fermentation (P4) (Supplementary Table S5) [61, 62]. Route assignments were robust across thresholds between 0.70 and 0.80, whereas higher thresholds generated route-specific annotation artifacts (Supplementary Fig. S19); results are therefore reported at ≥0.80. Each provider MAG was assigned to its dominant route using a composite score combining pathway completeness and genomic investment, whereas MAGs encoding near-equivalent routes were additionally classified as multi-route providers (Supplementary Table S19). Propionate pathways were scored using a threshold of 0.85, methanogenesis pathways (KEGG M00357/M00567) at 0.65, and glycolysis, acetate, and lactate production at 0.75. Lactate producers were restricted to MAGs encoding homo- or heterolactic fermentation, whereas mixed-acid fermentation was classified under acetate production. KO assignments were derived primarily from DRAM annotations with EC- and keyword-based fallback strategies (Supplementary Information).

Within-community functional redundancy (FRIa) was quantified using normalized abundance-weighted Shannon entropy, and between-community redundancy (FRIb) as Bray–Curtis dissimilarity of abundance-weighted provider profiles (FRIb = 1—Bray–Curtis dissimilarity) [63]. Full definitions of metabolic routes, KO sets, and redundancy metrics are provided in Supplementary Table S5.

## Results and discussion

### Early historical imprint gives way to environment-driven community organization

To assess how historical contingency and environmental constraints jointly shape AD communities, we first characterized temporal changes in community composition during serial enrichment. Shannon index declined sharply for both bacterial and archaeal communities (SI Fig. 2), with the strongest loss occurring between the inoculum and week 1, followed by stabilization by week 8. Diversity loss was consistently greater at pH 5.5 than at neutral pH, and was more pronounced for bacterial than for archaeal communities, consistent with stronger early viability filtering under acidic conditions. Diversity at week 8 was significantly lower than in the starting inoculum and remained significantly lower than at week 1 (paired Wilcoxon tests; BH-FDR correction).

**Figure 2:**
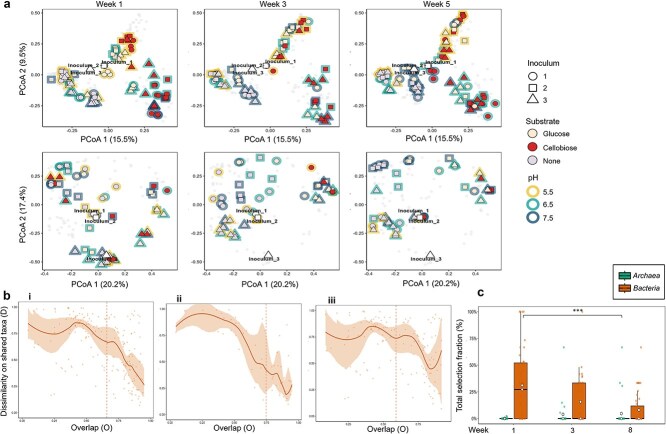
Temporal shifts in microbial diversity and community structure across substrates and pH gradients. (a) Principal coordinates analysis (PCoA) of Bray–Curtis dissimilarities showing temporal trajectories of bacterial (top) and archaeal (bottom) community composition across weeks 1, 3, and 8 based on 16S rRNA gene profiles. White diamonds denote inoculum communities. (b) DOA for bacterial communities at week 8. Curves show the relationship between overlap (O, shared relative abundance) and dissimilarity (D, Bray–Curtis dissimilarity on shared taxa) for: (i) all pairs within the same environment (i.e. pH and substrate), (ii) within-inoculum pairs, and (iii) between-inocula pairs*.* Shaded areas represent 95% bootstrap confidence intervals. (c) Temporal change in inferred assembly process fractions**.** Temporal decrease in the fraction of communities assembled by environmental selection (total = homogeneous + variable selection) in bacterial and archaeal consortia. Boxplots represent distributions across all substrates and pH conditions. Asterisks denote significant week 1–8 differences (Wilcoxon test, *P* value = .00039).

We next examined how inoculum history and environmental constraints jointly shaped community composition over time. Ordination analyses revealed a progressive reorganization of communities across transfers (Fig. 2a). At week 1, samples clustered primarily by inoculum identity, indicating a strong historical imprint. By week 3, overlap among inocula increased, and by week 8, communities segregated mainly by pH and substrate rather than by inoculum origin, indicating a shift toward environment-driven organization whereas historical differences were attenuated but not eliminated.

DOA at the final time point revealed partial, but incomplete, reproducibility of community composition under shared environmental conditions (Fig. 2b). Within the DOA framework, a negative relationship between taxonomic overlap and community dissimilarity supports reproducible assembly under shared environmental constraints, without implying strict convergence to a single taxonomic state. Communities cultivated under identical pH and substrate regimes exhibited a clear negative dissimilarity-overlap relationship (Fig. 2b-i), suggesting structured, non-random assembly. However, this relationship was consistently stronger for comparisons within the same inoculum than between different inocula (Fig. 2b-ii–iii), indicating that inoculum-specific signatures persist despite strong environmental filtering. Consistent with this pattern, permutational analysis of variance (PERMANOVA on Bray–Curtis dissimilarities; 9999 permutations) confirmed a progressive strengthening of environmental control over community composition through time. Environmental structuring was already detectable by batch 3 (c.a. 11 generations), and by week 8 (c.a. 30 generations) pH explained the largest fraction of compositional variance (*R*^2^ = 0.214, *P* = .001), followed by substrate identity (*R*^2^ = 0.04, *P* = .048), whereas inoculum identity was no longer significant (*R*^2^ = 0.035, *P* = .466).

To link compositional shifts to assembly mechanisms, we quantified the relative contributions of deterministic and stochastic processes through time. Dispersal limitation dominated assembly for both bacterial and archaeal communities throughout the experiment (Fig. 2c; SI Fig. 3), consistent with the closed nature of the enrichment system. In bacterial communities, homogeneous selection was detectable early but declined over time, whereas archaeal communities were largely governed by dispersal limitation across all sampling points. Other processes, including variable selection, drift, and homogenizing dispersal, contributed minimally. At week 8, we further examined whether the magnitude of selection-driven assembly covaried with independent physicochemical stress proxies measured in each microcosm, following a framework adapted from recent work [26]. The estimated fraction of deterministic assembly showed the strongest associations with short-chain carboxylate accumulation metrics, including total and undissociated SCCAs. Detailed analyses and statistical results are provided in the Supplementary Information.

**Figure 3:**
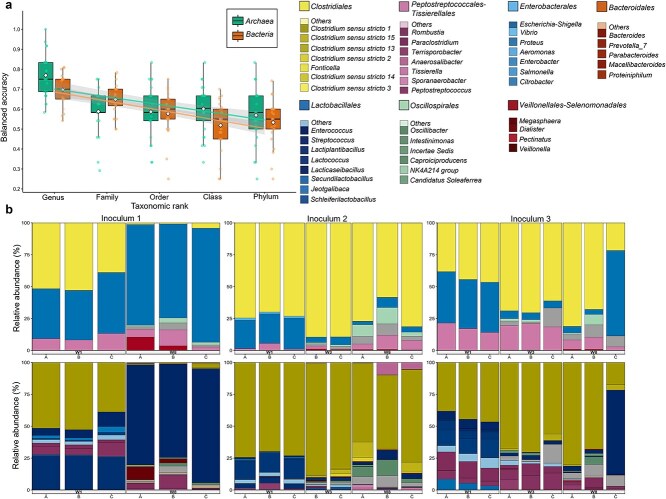
Taxonomic structure of bacterial and archaeal communities across hierarchical ranks at week 8. (a) Predictability of inoculum identity across hierarchical taxonomic ranks at week 8. Random forest classification of inoculum origin was performed independently for bacterial and archaeal communities at week 8 across hierarchical taxonomic levels (genus to phylum). Boxplots represent the distribution of balanced accuracies (macro recall) across repeated cross-validation folds. Regression lines (±95% CI) highlight the decline in predictability as communities are coarsened to higher ranks, indicating progressive taxonomic convergence. (b) Temporal dynamics of bacterial community composition across successive transfers. Stacked bar plots show the temporal evolution of bacterial community composition across transfers for one representative condition: cellobiose at pH 7.5. Taxonomic profiles are shown at the order level (top) and genus level (bottom). W1, W3 and W8 correspond to batches numbers. Only samples retaining ≥10 000 reads after quality filtering and chimera removal are shown to ensure sampling well above the saturation threshold for bacterial ASV richness. Additional community profiles across conditions and replicates are provided in Supplementary Figs S4, S5, S6, S7, S8, S9, S10, S11, S12, S13, S14, S15 and S16.

Together, these results reveal a progressive shift from historically structured assemblages toward configurations increasingly constrained by environmental conditions, without full taxonomic convergence. Early transfers were dominated by strong viability filtering (Fig. 2c, Supplementary Fig. S3), most pronounced under acidic conditions, leading to rapid diversity loss (Supplementary Fig. S2), a pattern also reported in other serial enrichment studies [64]. As enrichment proceeded, environmental parameters, most significantly pH, and to a lesser extent substrate, became the primary axes structuring community composition, whereas inoculum-specific signatures remained detectable at finer taxonomic resolution. At the final time point, supplementary analyses further indicated that the relative contribution of selection varied along physicochemical stress gradients associated with metabolite accumulation. DOA analyses corroborated this pattern, showing partial reproducibility under shared environments alongside persistent historical contingency, as communities derived from the same inoculum remained more similar than those assembled from different inocula [20]. Together, these findings align with assembly frameworks in which deterministic environmental filters constrain community composition without imposing a unique taxonomic outcome [65, 66].

Because residual contingency persisted despite strong environmental structuring, we next asked whether convergence emerges preferentially at coarser phylogenetic scales by quantifying how predictable inoculum identity remains across hierarchical taxonomic ranks under shared cultivation conditions.

### Scale-dependent taxonomic convergence under shared environmental constraints

To quantify convergence of taxonomic organization across distinct inocula under identical environmental conditions, we used random forest classification to predict inoculum identity from community composition at successive taxonomic ranks. In this framework, higher classification accuracy indicates stronger retention of inoculum-specific taxonomic structure, whereas declining accuracy reflects increasing convergence across inocula. Classification accuracy declined monotonically and significantly from genus to phylum for both bacterial (β = −0.046, *P* = 8.01 × 10^−11^) and archaeal communities (β = −0.039, *P* = 6.72 × 10^−6^) at week 8 (Fig. 3a), indicating progressive erosion of inoculum-specific signatures with increasing taxonomic coarsening. Although accuracy remained above random expectation at the phylum level, the steepest decline occurred between genus and family, indicating substantial loss of inoculum-specific information at intermediate ranks. By week 8, replicate cultures originating from distinct inocula showed substantial convergence at the order-level, most often dominated by *Clostridiaceae* (typically ~50%–60% of bacterial relative abundance), with recurrent secondary contributions from *Lactobacillaceae, Oscillospiraceae*, and *Peptostreptococcales*-*Tissierellales* across conditions (Fig. 3b; Supplementary Figs S6, S7, S8, S9, S10, S11, S12, and S13). Alternative stable configurations were observed in some cases, such as in inoculum 1 (Fig. 3b), where *Lactobacillaceae* rather than *Clostridiaceae* became dominant. Despite this convergence at the order level, genus-level bacterial profiles remained distinct among inocula (Fig. 3b, Supplementary Fig. S14), revealing finer-scale divergence within shared higher-level structures. This is particularly evident under glucose at pH 7.5, where communities with similar order-level composition display clearly differentiated genus-level assemblages (Supplementary Fig. S14). Taxonomic convergence was strongest under acidic conditions (pH 5.5; Fig. 3a, Supplementary Figs S15 and S16), consistent with tighter viability constraints that reduce taxonomic breadth under these conditions. Archaeal communities exhibited similar but more pronounced convergence patterns than bacterial communities, with reproducible community structures already emerging at the family- and, in some cases, genus-level across identical environments (Fig. 3a).

The scale-dependent convergence mirrors patterns reported in controlled microbial assembly experiments [19], in which reproducibility emerged at coarse taxonomic resolution (family level) despite persistent variability at finer scales. Consistently, our results show that enrichment under shared physicochemical constraints yields reproducible community organization at coarse taxonomic levels, whereas fine-scale taxonomic composition remains variable even after prolonged enrichment. Together, these findings reinforce the view that convergence toward similar metabolic regimes does not require taxonomic convergence, a decoupling widely documented across microbial systems [13, 66–68]. Such patterns are consistent with functional redundancy operating at higher taxonomic levels, where related taxa share overlapping metabolic capabilities yet differ in substrate range, energetic strategies, or interaction potential [69–71]. This raises the central question of whether predictable functional states in complex microbiomes are implemented through conserved functional repertoires within taxonomic lineages, or through alternative genetic solutions distributed across distinct taxa. To address this, we next quantified functional redundancy and provider turnover, focusing on how metabolic potential is partitioned across taxa assembled under identical environmental conditions.

### Functional redundancy and deterministic recruitment of metabolic roles

To quantify functional redundancy and accounting for both taxonomic composition and relative abundance, we used two complementary indices. The within-community functional redundancy index (FRIa) captures how evenly a given function is distributed among taxa within a community, whereas the between-community functional redundancy index (FRIb) quantifies whether communities assembled under identical conditions recruit the same taxa to perform that function. Together, these indices distinguish within-community redundancy from between-community convergence in functional provider composition.

**Figure 4:**
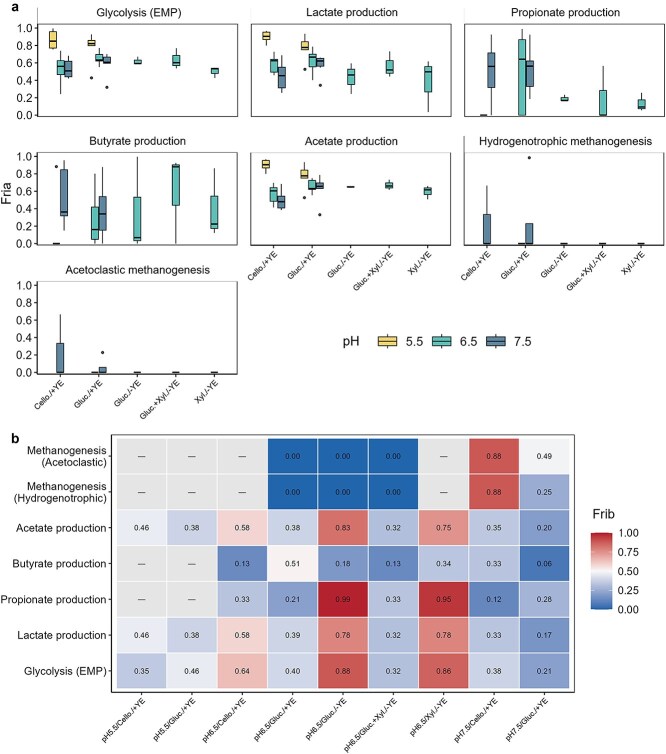
Within- and between-community functional redundancy across environmental conditions. (a) Within-community functional redundancy index (FRIa) for eight functional modules across substrates and pH. (b) Between-community redundancy index (FRIb) indicating convergence or divergence of functional providers under shared environments. Gluc: glucose, Xyl: xylose, Cello: cellobiose, YE: yeast extract.

FRIa revealed pronounced pathway-specific contrasts in how metabolic functions were distributed among taxa (Fig. 4a). Across cultivation regimes, functional redundancy declined consistently from upstream fermentative processes to terminal methanogenic functions, a pattern confirmed by Kruskal–Wallis tests across conditions (Supplementary Table S7). Glycolysis, acetate, and lactate production showed the highest redundancy overall (median FRIa 0.60–0.86), propionate and butyrate production intermediate redundancy, and methanogenic pathways near-zero redundancy. Redundancy for fermentative functions was also significantly higher at pH 5.5 than at near-neutral pH (Kruskal–Wallis; EMP *P* = 5.4 × 10^−4^, acetate *P* = 1.7 × 10^−4^, lactate *P* = 2.2 × 10^−4^; Supplementary Table S7). This pattern is consistent with strong compositional filtering under acidic conditions: at pH 5.5, where methanogenesis was absent and carbon flow was restricted to fermentation, the residual acid-tolerant community distributed glycolytic and lactogenic capacity more evenly across its members. In contrast, propionate redundancy decreased at pH 5.5, consistent with the near-absence of propionate production under acidic conditions, whereas methanogenic functions remained minimally redundant across all environments.

FRIb quantified the degree to which the same providers were recruited across replicate communities exposed to identical conditions (Fig. 4b). Fermentative functions showed the highest between-community convergence overall (FRIb mean 0.35–0.87), whereas methanogenic pathways were substantially more variable (FRIb mean 0.16–0.49), suggesting stronger stochastic or historically contingent assembly of methanogenic guilds. At pH 6.5, glycolysis and SCFA pathways showed relatively high convergence under single-substrate +YE conditions (0.48–0.52), whereas mixed substrates reduced convergence (0.32), consistent with increased availability of alternative taxonomic solutions. In several cases, -YE conditions were associated with higher FRIb values, suggesting that nutrient limitation may favor more taxonomically convergent functional solutions. Formal statistical tests did not detect significant differences in FRIb across conditions for any pathway (Kruskal–Wallis, all *P* > .4; Supplementary Table S8); FRIb patterns are therefore reported descriptively.

Together, these results indicate that functional stability in ad reflects a hierarchical organization of redundancy structured by pH. Upstream fermentative functions combined high within-community redundancy with moderate between-community convergence, particularly under acidic conditions where dominant fermenters concentrated shared functions at high abundance. Propionate and butyrate production occupied intermediate positions, whereas terminal methanogenic functions showed near-absent within-community redundancy and lower convergence across communities (Fig. 4), consistent with tighter energetic constraints and more stochastic assembly of methanogenic guilds (Fig. 2c) [7, 13, 40, 66, 72, 73]. Substrate-associated variation in convergence was most apparent at near-neutral pH, where multiple taxonomic solutions co-occurred within the same functional regime [40, 72]. More broadly, these patterns suggest that environmental selection acts hierarchically: pH first constrains the set of sustainable metabolic functions and subsequently shapes the diversity of taxonomic solutions capable of implementing them.

Because redundancy indices capture the distribution and recurrence of functional providers rather than the functional states achieved, we next integrated metabolite profiles with gene-level patterns to delineate pH-structured functional regimes across inoculum-environment combinations.

### Environmental filtering defines functional regimes

**Figure 5:**
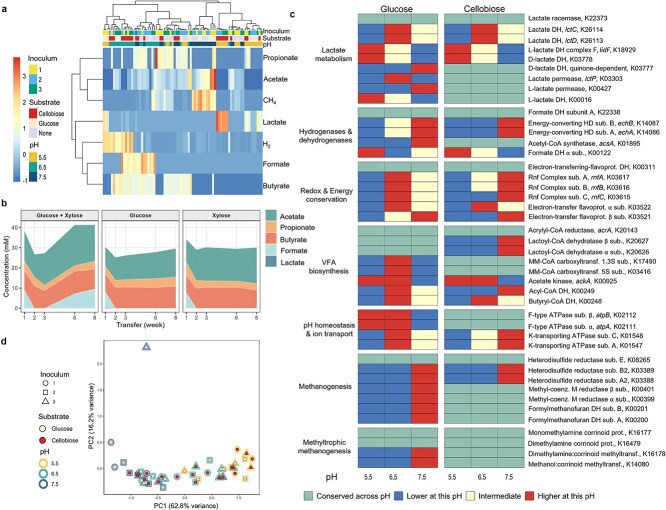
Metabolite profiles and gene-level functional patterns across pH and substrate conditions. (a) Hierarchically clustered heatmap of fermentation metabolite profiles across experimental conditions. Metabolite concentrations were standardized as z-scores across samples (centered and scaled per metabolite) to highlight relative enrichment patterns independent of absolute concentration differences. (b) Time-dependent establishment of fermentation end-product profiles in glucose, xylose, and mixed-sugar enrichments. Values represent the mean of three biological replicates, measured at the end of each batch. (c) Gene-level functional associations with pH. For each KEGG ortholog (KO), annotations indicate whether the gene shows its higher relative abundance, lower relative abundance, an intermediate response, or no statistically supported change across the pH gradient, based on effect sizes from linear models fitted to log-transformed data. Analyses were performed separately for glucose-amended microcosms, and cellobiose-amended microcosms, to identify pH-dependent trends shared across fermentable substrates. Prior to model fitting, near-absent KOs were filtered using a minimum abundance threshold of 1 × 10^−5^ and a minimum prevalence threshold of 5% of samples within each contrast. Only KOs showing statistically supported pH-associated contrasts (FDR-corrected *Q* < 0.05) are annotated as higher or lower at a given pH. KOs labeled as intermediate display effect sizes that fall between the lower and higher pH extremes, whereas KOs labeled as conserved across pH did not exhibit statistically supported differences across pH conditions. For clarity, the heatmap displays a curated subset of functionally relevant genes; complete results for all tested KOs are provided in Supplementary Table S6. (d) PCoA based on Bray–Curtis dissimilarities of KO abundance profiles, illustrating similarities in functional gene composition across samples under shared pH and substrate regimes. VFA: Volatile fatty acid, e.g. propionate, butyrate, and formate. All analyses presented in this figure are based on enrichment microcosms derived from inocula 1–3. H_2_: dihydrogen, CH_4_: methane, PC: principal component, MM: methylmalonyl, prot.: protein, sub.: subunit, DH: dehydrogenase, transf.: transferase, coenz.: coenzyme.

To characterize the functional states achieved under shared environmental conditions, we quantified metabolite production across pH and substrate treatments after eight weekly transfers. Communities clustered primarily by pH rather than inoculum or substrate (Fig. 5a; Supplementary Table S15). Acidic conditions (pH 5.5) were characterized by lactate accumulation and negligible methane production, whereas near-neutral pH (7.5) favored methanogenesis with limited accumulation of soluble intermediates, primarily acetate. Intermediate pH yielded mixed acidogenic-methanogenic profiles, including longer-chain SCCAs such as butyrate. Glucose and cellobiose produced broadly similar pH-dependent metabolic states, differing mainly in quantitative rather than qualitative profiles. To examine how these states emerged over time, we tracked volatile fatty acid (VFA) dynamics across successive transfers in glucose, xylose, and mixed-sugar enrichments (inoculum 4; Fig. 5b; Supplementary Table S16). From transfer 3 onwards in glucose and xylose conditions, and from transfer 6 in mixed substrates, end-point metabolite profiles stabilized across transfers, indicating reproducible convergence toward condition-specific fermentation states independent of transfer number.

Gene-level enrichment analyses revealed strong pH-dependent structuring of ad functions (Fig. 5c), most pronounced for downstream redox-balancing and terminal electron-acceptors pathways, whereas genes associated with central carbohydrate metabolism and glycolysis showed little pH-dependent variation within substrate contexts. Lactate metabolism displayed a clear partitioning along the pH gradient (Fig. 5c; Supplementary Table S6): fermentative lactate-production genes, including L-lactate dehydrogenase (K00016), auxiliary L-LDH subunits (K18929), and NAD-dependent D-lactate dehydrogenase (K03778), were enriched at pH 5.5, whereas lactate uptake and oxidation functions dominated at near-neutral pH (6.5–7.5). In particular, ferredoxin-coupled lactate oxidase subunits (K26113/K26114) were consistently enriched at higher pH across both glucose and cellobiose conditions, whereas quinone-dependent D-lactate dehydrogenase (K03777) and lactate permeases (K00427, K03303) were enriched mainly under glucose at pH 6.5–7.5. Downstream SCFA-production pathways showed similar structuring: butyryl-CoA dehydrogenase (K00248) and acyl-CoA dehydrogenase (K00249) were enriched at near-neutral pH across both substrates, whereas lactoyl-CoA dehydratase subunits (K20626/K20627), associated with lactate channeling into propionate via the acrylate pathway, were specifically enriched at pH 7.5 in cellobiose communities. Electron-accepting pathways were similarly reorganized, with formate dehydrogenase (K00122) enriched at pH 5.5, whereas electron-transfer flavoproteins (K03522/K03521), the Rnf complex (K03617/K03616/K03615), and energy-converting hydrogenase subunits (K14086/K14087) were enriched at near-neutral pH, consistent with increased reliance on electron bifurcation, ferredoxin oxidation, and interspecies hydrogen transfer under syntrophy-permissive conditions. Methanogenesis-associated genes followed the same pattern: core hydrogenotrophic methanogenesis functions, including methyl-coenzyme M reductase (K00399/K00401), formylmethanofuran dehydrogenase (K00200/K00201), and heterodisulfide reductase (K03388/K03389), together with methylotrophic methanogenesis genes such as methanol:corrinoid methyltransferase (K14080) and dimethylamine:corrinoid methyltransferase (K16178), were enriched primarily at pH 7.5 under glucose. Conversely, genes associated with pH homeostasis and ion transport, including F-type ATPase subunits (K02111/K02112) and potassium-transporting ATPase subunits (K01547/K01548), were enriched at pH 5.5. Together, these patterns indicate that pH restructures ad metabolism primarily through downstream electron flow and terminal electron-accepting pathways rather than upstream carbohydrate-processing functions.

Multivariate analysis of gene patterns across samples further supported pH-structured functional convergence (Fig. 5d). Replicate communities seeded from distinct inocula occupied overlapping regions of ordination space within each pH condition, whereas communities sharing inoculum but exposed to different pH conditions diverged. Substrate identity modulated functional composition within pH bands but did not override pH-driven structure.

Together, these patterns indicate convergence toward comparable functional regimes across distinct starting consortia exposed to shared physicochemical constraints, although responses differed across metabolic layers. Genes associated with redox balancing and terminal electron-accepting functions exhibited the strongest pH-dependent structuring, whereas upstream carbohydrate-processing functions remained comparatively stable, consistent with environmental filtering acting most strongly on energetically constrained electron-flow processes [72, 74]. Accordingly, methanogenesis-associated functions were enriched at neutral to mildly alkaline pH, whereas acidic conditions favored alternative electron sinks, including lactate- and formate-associated pathways and VFAs [35, 75]. The reciprocal enrichment of lactate production versus utilization across the pH gradient further suggests pH-dependent reorganization of cross-feeding interactions, with low pH favoring reduced end-product accumulation and higher pH supporting tighter metabolic coupling through lactate uptake and oxidation [76]. Enrichment of F-type Together, metabolite- and gene-level patterns indicate that pH reproducibly structures downstream redox-balancing and terminal electron-sink functions across communities. However, these analyses primarily define the functional states reached, rather than how they are implemented taxonomically. We therefore next performed a pathway-resolved analysis to quantify metabolic network architecture and its taxonomic realization across environmental regimes.

### Environmental selection constrains metabolic network architecture despite taxonomic variation

**Figure 6:**
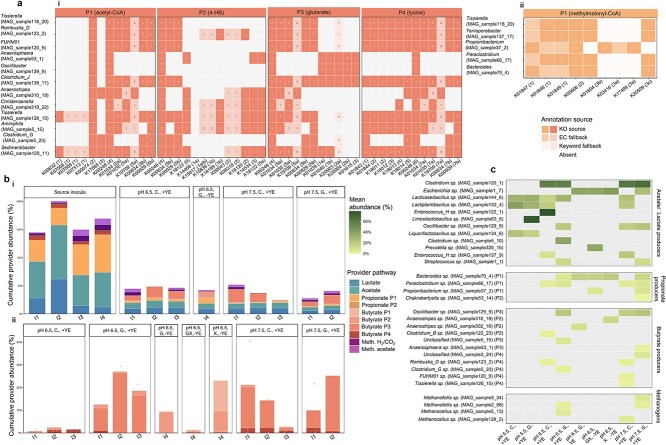
Metabolic pathway architecture and taxonomic composition of fermentation provider guilds across pH and substrate conditions. (a) Key enzyme distribution (KO heatmap) across representative MAGs for butyrate (P1–P4) and propionate (ii, P1) producers. Each cell indicates gene copy number; shading reflects annotation confidence (KO direct assignment vs. EC fallback). MAGs were selected as the highest-abundance representatives of each route based on genome-resolved metagenomics. (b) Cumulative provider abundance for main functions and putative butyrate producers across conditions. (i) Stacked bars show the cumulative relative abundance of MAGs encoding each metabolic function (lactate, acetate, propionate, butyrate, methanogenesis), averaged across replicates. (ii) Bars show the mean cumulative relative abundance of butyrate-producing MAGs assigned to each biosynthetic route (P1–P4), averaged across all replicates. Points represent individual replicates in which at least one butyrate provider was detected. (c) Relative abundance of pathway provider MAGs across all enrichment conditions, grouped by dominant metabolic route and colored by mean abundance. Mean abundance is calculated across replicate samples where the MAG was detected (relative abundance >0); conditions where the MAG was absent are shown in grey. YE: Yeast extract, I: inoculum, G.: glucose, X.: xylose, C.: cellobiose, GX: glucose + xylose, Meth.: methanogenesis.

We next characterized metabolic network architecture at the pathway level across communities assembled under identical cultivation conditions. Network architecture was defined as the genomic representation and coupling of alternative metabolic pathways routing carbon and electrons from central metabolism toward fermentation products and terminal electron sinks, as inferred from genome-resolved metagenomics. Pathway representation was quantified using complementary abundance-, copy-number-, and provider-based metrics (Supplementary Tables S9, S10, S11, S12, and S13). For butyrate and propionate, an additional genomic investment score quantified relative allocation across alternative routes (Supplementary Tables S16, S17, S18, and S19). Representative MAGs and diagnostic KO profiles are shown in Fig. 6a. Hereafter, “providers” refers to MAGs exceeding the pathway completeness threshold and therefore inferred to encode the corresponding metabolic function (Methods).

All four inocula encoded genomic potential for the major fermentation functions, although the relative abundance of putative providers differed substantially (Fig. 6b-i). Propionate-provider abundance was consistently high in source communities (24%–54%), whereas butyrate providers were lower and more variable (3%–22%). Multiple butyrate and propionate routes were detectable in inocula, often through low-abundance MAGs with partial pathway completeness (Supplementary Tables S9–S12), providing distinct functional reservoirs subsequently filtered by enrichment conditions. Enrichment primarily restructured these pre-existing capacities through selective amplification and loss rather than emergence of novel pathways. This was most evident for propionate, where provider abundance collapsed across enrichments (1.3%–8.2%) and dominant taxa shifted entirely relative to the inocula, from *Cloacimonas*-dominated assemblages to *Bacteroides, Paraclostridium*, and *Propionibacterium*. Across enrichment cultures, pathway architecture was strongly structured by pH: upstream carbohydrate-processing functions, including glycolysis and acetate production, remained broadly distributed and taxonomically redundant (glycolysis provider Shannon H′ 1.0–1.6; Supplementary Fig. S17), whereas downstream fermentative functions showed stronger condition-dependent filtering.

Lactate production occurred across all pH conditions but was implemented by distinct taxonomic guilds depending on environmental context (Fig. 6c). At pH 5.5, lactate production was dominated by acid-tolerant *Lactobacillaceae*, whereas near-neutral communities recruited more diverse provider assemblages including *Clostridium, Escherichia, Bifidobacterium*, and *Enterococcus_H* depending on substrate and yeast extract (YE, -YE/+YE conditions) supplementation. Despite substantial taxonomic turnover, pathway representation remained stable within conditions, consistent with functional redundancy across phylogenetically distinct taxa. Furthermore, across conditions where propionate was detected (pH 6.5 and 7.5), the methylmalonyl-CoA pathway (P1) was the overwhelmingly dominant route, accounting for >95% of propionate provider abundance across inocula and conditions (Fig. 6a-ii, Supplementary Fig. S18). A minor propanediol/acrylate pathway contribution, carried by *Chakrabartyella*, was detected sporadically at low abundance without condition-dependent enrichment. Route assignment was supported by consistent co-occurrence of diagnostic pathway genes in provider MAGs (Fig. 6a; Supplementary Table S17), whereas extracellular succinate remained low (Supplementary Table S14), consistent with rapid turnover of this intermediate.

In contrast, butyrate production deployed different biochemical routes depending on both pH and YE availability, as assessed by cumulative provider abundance (Fig. 6b-ii) and investment-weighted route allocation (Supplementary Fig. S18). Under +YE conditions, the glutarate pathway (P3) dominated butyrate-provider biomass in the most productive communities, reaching 10%–27% cumulative abundance (e.g. pH 6.5 + YE glucose I2: 26.8%; pH 7.5 + YE cellobiose I1: 20.6%; pH 7.5 + YE glucose I1-I2: 10%–13%). In route-allocation terms, P3 accounted for 55%–99% of the provider pool and was carried primarily by *Oscillibacter, Clostridium_J,* and *Vermiculatibacterium*. The lysine fermentation pathway was also dominant in a few conditions, including cellobiose-amended communities at pH 6.5 under YE supplementation. A few MAGs contributed to multiple routes including *Clostridium_J,* which qualified simultaneously as a P3 + P4 provider, and *Anaerostipes* which contributed to both P1 and P2 pools (Fig. 6a, Supplementary Information). The lysine fermentation pathway (P4), carried by *JAVXAG01, Romboutsia_D*, and *Tissierella*, consistently co-occurred with P3 under +YE at pH 7.5, contributing 16%–52% of provider abundance and 20%–43% of route allocation, and became the sole detected route in I3 under pH 6.5 + YE cellobiose. The 4-hydroxybutyrate pathway (P2) also contributed substantially under pH 6.5 + YE cellobiose in I1 and I2, where it dominated route allocation despite modest total provider abundance (0.8%–1.6%). Under -YE conditions, the dominant butyrate signal shifted toward P2 dominance, particularly under xylose. P2-encoding providers reached 9.5% cumulative community abundance, with individual replicates reaching 14%, ~10-fold higher than under any +YE condition (mean 0.8%, max 2.1%; Fig. 6c). This enrichment is mechanistically consistent with succinate production during pentose catabolism, which can fuel the GABA/4-hydroxybutyrate entry route independently of exogenous amino acid supply. In contrast, P3-mediated capacity remained detectable but strongly reduced: on glucose -YE, P3-encoding organisms reached only 3.3% cumulative abundance versus 10%–27% under +YE glucose conditions. This +YE/-YE contrast is mechanistically coherent with route biochemistry. P3 and P4 are amino-acid-associated pathways requiring glutamate-derived intermediates or lysine, respectively, explaining their enrichment under amino acid supplementation. By contrast, P2 can draw on succinate- or GABA-linked intermediates derived directly from central carbon metabolism, allowing it to become dominant when exogenous amino acids are absent. Residual low-level detection of amino-acid-associated routes under -YE conditions may additionally reflect endogenous amino acid release through cell lysis during batch cultivation.

Despite taxonomic variability, pathway-level organization was highly reproducible within environmental conditions. Propionate route structure was nearly invariant across inocula and replicates, whereas butyrate route allocation and provider abundance patterns were largely reproducible within conditions, both across replicates and across inocula. For butyrate, P3 dominated independently across I1, I2, and I3 under pH 6.5 + YE glucose, and across detected inocula under pH 7.5 + YE glucose and cellobiose. Within individual inocula, intra-replicate variability was low where multiple replicates were detected: for example, P3 allocation under pH 7.5 + YE I2 cellobiose was 88.7%, 89.1%, and 88.1% across the three replicates, and exceeded 96% in all detected replicates under pH 6.5 + YE glucose for I2 and I3. Where variability was higher, such as I3 cellobiose pH 7.5, where P3 ranged from 44%–58% and P4 from 31%–43% across two replicates, reflecting route co-dominance. The main inter-inoculum divergence occurred under pH 6.5 + YE cellobiose, where I1 and I2 were P2-dominant whereas I3 was P4-dominant. Overall, dominant route identity remained highly robust across biological replicates and inocula sharing the same environmental conditions, supporting deterministic environmental filtering rather than stochastic assembly.

Taken together, these findings indicate that equivalent metabolic functions can be implemented by different taxa provided that the underlying genetic potential is conserved, supporting a trait- and network-based view of functional guilds [7, 66, 77, 78] Across independent inocula, communities reproducibly converged toward similar pathway-level architectures despite persistent taxonomic variability, indicating that environmental selection acts more strongly on metabolic organization than on species identity itself. Collectively, our results support a hierarchical model of community assembly in which pH acts as the primary environmental gate constraining feasible metabolic network architectures, whereas taxonomic composition remains comparatively flexible within those constraints. Mechanistically, pH alters both the energetic feasibility of metabolic reactions and the ecological interactions they sustain. Because reactions involving proton exchange shift by n × 5.7 kJ mol^−1^(where n is the number of protons exchanged) in ΔG′ per pH unit [79, 80], near-equilibrium syntrophic oxidations such as propionate and butyrate conversion to acetate, CO_2_, and H_2_ become strongly constrained under acidic conditions [74, 79]. At the same time, acid-tolerant fermenters, including lactate producers, gain competitive advantages at low pH [76, 81], whereas methanogens are inhibited [75]. Beyond these direct physiological effects, pathway selection also reshapes the metabolite landscape available for cross-feeding: by constraining which fermentation intermediates and electron carriers are produced, pH indirectly restructures the trophic interactions that can be maintained within the community. Although metabolite accumulation remained below reported inhibitory thresholds [82], intermediate production is intrinsically coupled to pathway activity and can still reshape interaction networks by modulating substrate availability for downstream guilds. Community organization therefore reflects both direct physicochemical constraints on pathway feasibility and the emergent metabolite environment generated by those constraints.

Within this framework, selection among alternative pathways appears to reflect trade-offs among thermodynamic feasibility [74, 79], kinetic performance [83], regulatory control [81], and physiological regulation [18]. Under the batch cultivation regime used here, retention of specific pathway configurations likely reflects selection across transfer cycles favoring strategies that maximize fitness over the transfer interval, consistent with observations from synthetic consortia [18]. The tested substrates enter central metabolism through relatively conserved carbohydrate-processing routes, limiting generalization of upstream pathway diversity. More chemically distinct substrates, such as proteins, nucleic acids, or long-chain fatty acids, would likely expand the accessible pathway solution space and engage different redox chemistries [64, 84]. Yeast extract supplementation further modulated butyrate route deployment by favoring amino-acid-associated pathways and broader shifts in resource allocation, including enrichment of sporulation-associated markers and reduced biosynthetic investment (Supplementary Table S18). However, these effects primarily altered which biochemical routes were deployed within the accessible functional space rather than overriding the broader pH-dependent organization of terminal electron-sink pathways. Regardless of substrate entry route, pH-dependent thermodynamic constraints ultimately act on shared downstream conversions which remain governed by the same feasibility landscape across substrate types because central carbon metabolism converges on common intermediates subject to identical pH- and H_2_-partial-pressure-dependent constraints [85]. We therefore expect pH-dependent structuring of terminal electron-sink pathways to extend beyond the carbohydrate regime tested here, although acknowledging that chemically distinct substrates would likely modulate which alternative pathways are selected within that constrained functional space [84].

Finally, our analyses focus on genomic potential, delineating the space of feasible metabolic interactions. A further limitation inherent to genome-resolved metagenomics is that catabolic and anabolic routes for a given metabolite sometimes share enzymatic machinery—for instance, some butyrate-associated pathways are reversible, meaning that organisms annotated with butyrate synthesis genes may in some contexts operate as consumers. Where such ambiguity arose, we resolved pathway directionality by reference to the literature, following established route definitions [61]. Consequently, all provider assignments in this study remain putative, because genomic potential does not constitute direct evidence of metabolic activity. Although metatranscriptomics would have provided additional resolution, particularly for MAGs encoding multiple complete routes simultaneously, the high reproducibility of provider abundance patterns across replicates and inocula, combined with the simplified and controlled substrate regimes used here, allowed us to reveal the dominant environmental structuring of metabolic network architecture.

## Supplementary Material

Supplementary_material_wrag145

## Data Availability

All data supporting the findings of this study, including raw data and metadata, have been made publicly available on ENA (Submissions: PRJEB108113, PRJEB114047, and PRJEB114111).
