# Supplementary material for: Environmental selection constrains metabolic network architecture despite taxonomic turnover in anaerobic digestion communities
Source: ISME J. 2026 Jun 8;20(1):wrag145. doi: 10.1093/ismejo/wrag145 (PMC13325402; doi:10.1093/ismejo/wrag145)
Supplement: Supplementary_material_wrag145 [file supplementary_material_wrag145.zip › SI_Information_and_Figures_v5_wrag145.docx]

# **Environmental selection constrains metabolic network architecture despite taxonomic turnover in anaerobic digestion communities**

**Authors:** Lisa Jourdain^1^, Aaron Leininger^1,2^, Alan R. Pacheco^3^, Wenyu Gu^1^*

1. MICROBE laboratory, Institute of Environmental Engineering, School of Architecture, Civil and Environmental Engineering, Swiss Federal Institute of Technology, Lausanne, Switzerland
2. Microbial Systems Ecology Laboratory, Department of Environmental Microbiology, Swiss Federal Institute of Aquatic Science and Technology, Zurich, Switzerland
3. Department of Fundamental Microbiology, University of Lausanne, Lausanne, Switzerland

## **Supplementary Information**

**Basal medium recipe:** All microcosms were prepared in a defined anaerobic basal medium containing: KH_2_PO_4_/K_2_HPO_4_ phosphate buffer, (NH_4_)_2_SO_4_ 0.3 g L^-1^, NH_4_Cl 2 g L^-1^, NaCl 0.5 g L^-1^, MgCl_2_ 0.15 g L^-1^, CaCl_2_·2H_2_O 0.1 g L^-1^, cysteine-HCl as reducing agent, resazurin (0.5 mL L^-1^ of a 0.1% solution) as redox indicator, modified Wolin’s mineral solution (10 mL L^-1^), and Wolin’s vitamin solution (0.5 mL L^-1^). Carbon source and nutrient supplementation were varied according to treatment. Carbon substrates were added as: no added carbon source, glucose (15 mmol L^-1^), xylose (15 mmol L^-1^), glucose + xylose (7.5 mmol L^-1^ each), or cellobiose (7.5 mmol L^--1^). Yeast extract was supplied at 0.2% (w/v) where indicated.

**Estimated number of generations across serial transfers:** We estimated the number of microbial generations per transfer from changes in optical density at 600 nm (OD_600_) between the start and end of each incubation interval, assuming exponential growth and binary fission. Let *OD_t,0_* and *OD_t,1_* denote OD_600_ at the beginning and end of transfer *t*, respectively, and let *f* be the transfer fraction (inoculated volume / total culture volume). The effective starting biomass proxy after dilution is *OD_t,0_^′^=f×OD_t−1,1_*, where *OD_t−1,1_*​ is the final OD of the preceding transfer. The number of generations during transfer *t* is then:

$$g{}_{t}={log}_{2}(\frac{{OD}_{t,1}}{{f\times OD}_{t,0}^{'}})$$

Total generations over a time window were computed as $G=\sum_{t} g_{t}$. This OD_600_-based estimate captures net population doublings implied by biomass increase and transfer dilution, but does not resolve differential growth among taxa or deviations from linearity between OD_600_ and biomass across conditions.

**Gas and volatile fatty acid measurements:** Headspace gas samples were collected from cultivation bottles using a gastight syringe. A 300 µL aliquot was injected into a gas chromatograph (SRI Multigas 5 TCD-FID with methanizer; SRI Instruments, USA) equipped with argon as carrier gas. Gas separation was performed on a 2 m Hayesep D column maintained at 80 °C. Gas concentrations were quantified using multi-point external calibration curves.

For liquid-phase analyses, 1.5 mL culture samples were withdrawn and filtered through 0.2 µm syringe filters into HPLC vials. Samples were injected into an Agilent Hi-Plex H column (7.7 × 300 mm, 8 µm; p/n PL1170-6830, Agilent Technologies, USA) maintained at 50°C. Separation was achieved using 5 mmol L^-1^ H_2_SO_4_ as the mobile phase at a flow rate of 0.6 mL min^-1^. Volatile fatty acids were quantified based on retention times and external calibration curves using a G7162A/B refractive index detector.

**pH dynamics during serial enrichment.** To evaluate pH stability across transfers, we monitored pH at the end of each 7-10 day incubation period. A uniform 100 mM phosphate buffer was applied across all pH regimes to maintain consistent ionic strength without introducing phosphate concentration as a confounding variable. Using standard buffer capacity theory (β = 2.303 × C × Ka[H^+^]/(Ka + [H^+^])^2^, with pKa_2_ = 7.2 for the H_2_PO_4_^-^/HPO_4_^2-^ couple), this yields β ≈ 4.4, 31.9, and 51.2 mmol L⁻¹ pH unit⁻¹ at pH 5.5, 6.5, and 7.5, respectively, reflecting the ~10-fold lower buffering capacity at pH 5.5 relative to pH 7.5. Complete homolactic fermentation of 15 mmol L^-1^ glucose would release approximately 30 mmol L^-1^ of protons (2 H^+^ per glucose, lactate pKa = 3.86). At pH 6.5 and 7.5, this implies theoretical maximum pH drifts of ~0.9 and ~0.6 units respectively under this worst-case scenario. At pH 5.5, the buffering capacity is substantially exceeded under this assumption; however, proton release is expected to be self-limiting through product-mediated inhibition of fermentative activity at low pH. In practice, observed within-transfer pH variation remained within 0.5-1.3 units across all conditions. The observed variation is unlikely to have substantially altered community structure for two reasons: transient drifts remained within the same physiological regime throughout (no condition crossed the ~pH 6.0-6.5 threshold separating methanogenic from acidogenic conditions [1, 2]), and the strong, reproducible clustering of community composition and metabolite profiles along the pH gradient, confirms that communities experienced and responded to distinct pH environments throughout enrichment.

**Assembly-process inference and community-convergence analysis:** Community assembly mechanisms were quantified by partitioning community turnover into deterministic and stochastic components [3]. Pairwise phylogenetic turnover was quantified using abundance-weighted βMNTD, and deviations from null expectations (999 randomizations) were summarized as βNTI. Values of |βNTI| > 2 were interpreted as evidence for deterministic selection (positive: heterogeneous; negative: homogeneous). For pairs with |βNTI| ≤ 2, compositional turnover was further assessed using Raup-Crick Bray-Curtis dissimilarity (RCbray), with values > 0.95 or < -0.95 indicating dispersal limitation or homogenizing dispersal, respectively, and intermediate values attributed to ecological drift. The relative contribution of each assembly process was summarized within and across inocula and treatments.

Reproducibility and convergence of community trajectories were assessed using Dissimilarity-Overlap Analysis (DOA) [4]. For each treatment, overlap in shared ASVs between replicate communities was related to Bray-Curtis dissimilarity. A negative dissimilarity-overlap relationship was interpreted as evidence for reproducible community organization under shared environmental conditions.

**Definition of metabolic routes:** Metabolic functions were operationally defined as discrete anaerobic digestion modules representing major carbon transformation steps, including glycolysis, short-chain fatty acid (SCFA) production, and methanogenesis. Functions were inferred at the MAG level from KEGG Ortholog (KO) annotations using curated, function-specific KO sets. A MAG was classified as a potential functional provider when it encoded the key diagnostic enzymes, or minimal enzyme combinations, required to support the corresponding metabolic conversion. For end products that can be generated via alternative biochemical routes, provider status was assigned if at least one complete and biologically plausible enzymatic configuration was detected. Metabolic routes and KO definitions were curated based on extensive literature review (see main text references) and are detailed below; they are also summarized in Supplementary Table S5.

- Butyrate production. Four biochemically distinct routes to butyrate were defined following previously established recommendations [5], each operationalized as a complete enzymatic sequence from carbon entry to the terminal CoA-transfer or phosphotransbutyrylase-butyrate kinase module.
  - P1: Acetyl-CoA condensation route (thiolase, 3-hydroxybutyryl-CoA dehydrogenase, crotonase, *bcd,* But or *ptb/buk*): KO set {K00632/K07508/K07509/K07513, K00074, K17865, K00248, terminal: (K01034 AND K01035) OR K19709 OR (K00634 AND K00929)}. The ETF subunits (*etfA/etfB*; K03521, K03522) associated with *bcd*-mediated electron transfer were intentionally excluded from pathway scoring. Although the *bcd*-ETF complex is important for energy conservation under low-potential conditions, *etfA/etfB* are frequently absent or misannotated in environmental MAGs, and their inclusion would artificially lower completeness scores for MAGs that are otherwise fully competent for butyrate production via P1. Their exclusion is consistent with approaches used in comparable metagenomics-based analyses [6].
  - P2: 4-Hydroxybutyrate/4-aminobutyrate route (*abfT*/GABA-CoA transferase entry, succinyl-CoA reductase, 4-hydroxybutyrate dehydrogenase, *abfD*/vinylacetyl-CoA isomerase, *bcd, 4Hbt* terminal or *ptb/buk*): KO set {K18122, K14468/K15017/K15038/K18119, K00043/K08318/K18120, K14534, K00248, terminal: K18122 OR (K00634 AND K00929)}. The scoring expression is anchored at the GABA/4-HB entry point, which is common to all P2 flux regardless of upstream carbon source.
  - P3: Glutarate route (*gct, hgCoAd, gcd, bcd, but* or *ptb/buk*): KO set {(K01039 AND K01040), (K20903 AND K20904), K01615, K00248, terminal: (K01034 AND K01035) OR K19709 OR (K00634 AND K00929)}. *gct* (glutaconate CoA-transferase; K01039/K01040) and *hgCoAd* (2-hydroxyglutaryl-CoA dehydratase; K20903/K20904) are heterodimeric complexes in which both subunits are required for activity; the scoring expression therefore uses AND logic for each pair rather than OR. The *hgCoAd* activator protein (HgdC/component A) was not included as a required KO: although biochemically necessary for initiating catalysis, it is expressed at very low concentrations in organisms known to harbor this pathway and is systematically underdetected in metagenomic annotations. *L2Hgdh* (2-hydroxyglutarate dehydrogenase), which converts 2-oxoglutarate to 2-hydroxyglutarate upstream of *gct*, was likewise excluded from the scoring expression because it is not universally required when glutarate itself or 2-hydroxyglutarate serves as the entry substrate, and because its annotation in KEGG is inconsistent across reference genomes.
  - P4: Lysine fermentation route (*KamA, KamD/E, Kdd, Kce, Kal, Bcd, Ato* or *Ptb/Buk*): KO set {K01843, K01844/K18011, K18012, K18013, K18014, K00248, terminal: (K01034 AND K01035) OR K19709 OR (K00634 AND K00929)}. *KamD/E* (β-lysine 5,6-aminomutase; K01844/K18011) share the same EC number (5.4.3.3) and are scored with OR logic, consistent with the EC-based fallback annotation strategy used in this pipeline and with the frequent annotation of only one subunit per MAG in environmental genomic datasets.
- Propionate production. Three routes were defined: P1, the methylmalonyl-CoA (succinate/fumarate) pathway, and P2, the acrylate pathway; and P3, the propanediol utilization pathway (propanediol dehydratase *PduCDE*, propionaldehyde dehydrogenase *PduP*, phosphate propanoyltransferase *PduL*, propionate kinase *PduW*). KO definitions follow curated sets as described above and in Supplementary Table S5. All three routes were evaluated systematically; P3 was detected at threshold in a limited number of conditions and is noted where relevant (Supplementary Table S5).
- Lactate, acetate, and glycolysis. Homolactic fermentation was defined by the presence of L- or D-lactate dehydrogenase (K00016/K03778). Heterolactic fermentation additionally requires 6-phosphogluconate dehydrogenase (K00033/*gnd*), shared with the phosphoketolase pathway; combined detection of K00033 with *ldh* was used as the defining signature. Acetate production was anchored on the phosphotransacetylase–acetate kinase module (*pta/ackA*; K00625/K13788 and K00925). EMP glycolysis was defined using a fixed KO set (see Supplementary Table S5) with phosphofructokinase (K00850/K16370) as a required gateway enzyme.
- Methanogenesis. Acetoclastic and hydrogenotrophic methanogenesis were scored using KEGG module definitions M00357 and M00567, respectively. Methylotrophic methanogenesis was defined as the union of the methanol-to-methane and methylamine-to-methane routes.

Completeness thresholds and KO definitions are provided in Supplementary Table S5.

**Multi-route butyrate provider assignment.**  Among the 76 MAGs classified as butyrate providers at a pathway completeness threshold of ≥ 0.80, 69 (91%) qualified exclusively for a single route, while 7 (9%) encoded two or more biochemically complete routes simultaneously — defined as routes whose pathway completeness scores differed by no more than 5 percentage points from the dominant route (Supplementary Table S19). In the main analysis, each MAG was assigned to its single dominant route using the argmax of the composite score (investment score × coverage (cov)), consistent with the expectation, established in gut microbiome analyses, that multi-route providers are uncommon at stringent completeness thresholds. For the 7 MAGs meeting the near-equal completeness criterion, provider abundance and route allocation were additionally computed with these MAGs counted in all qualifying routes. The 7 multi-route MAGs spanned seven genera: *Anaerostipes, Aminipila,* *Vermiculatibacterium, Otoolea, Clostridium_J, UBA5206*, and *Anaerosalibacter*. Pathway co-occurrence was taxonomically coherent across cases. Double qualification for P2 + P4 (4-hydroxybutyrate and lysine fermentation, both amino-acid-dependent entry points) was the most frequent combination (3 MAGs*: Aminipila, UBA5206, Anaerosalibacter*), consistent with the shared metabolic context of amino acid catabolism. *Clostridium_J* qualified for both P3 and P4 at maximum completeness (cov = 1.0 for both), consistent with published reports of dual glutarate/lysine fermentation capacity in some Clostridium species. *Anaerostipes* and *Vermiculatibacterium* each qualified for P1 + P2 (cov = 0.833/0.800 and 0.833/0.800 respectively), consistent with the documented metabolic flexibility of these *Lachnospiraceae* relatives, which can produce butyrate both via the acetyl-CoA condensation route and the 4-hydroxybutyrate/GABA route. Otoolea qualified for P2 + P3 (cov = 0.857/0.833), a co-occurrence consistent with GABA/glutamate catabolism feeding both pathways. The quantitative impact of multi-route assignment on community-level provider abundances was minor in all but one case. *Anaerostipes* (maximum community abundance 13.7%) was the only MAG with a quantitatively consequential dual assignment: on xylose -YE in I4, where it reaches its highest abundance, including it in both P1 and P2 introduced a Δ = +8.9 percentage points on. In all other conditions and for all other multi-route MAGs, the maximum difference between single-route and multi-route cumulative provider abundances was < 1.2 percentage points per route. Route-level conclusions were entirely unchanged under multi-route assignment, providing confidence that these patterns are not sensitive to the assignment strategy used for the small minority of MAGs with genuinely dual pathway potential.

**EC-based fallback and subunit resolution.** Where direct KO identifiers were absent from DRAM annotations, KO assignments were inferred from EC numbers extracted from functional descriptions using exact four-level EC matching. This approach captures a substantial fraction of functionally annotated genes that lack explicit KO assignments, which is common in environmental metagenomes. The EC fallback is inherently less specific than direct KO assignment for two reasons. First, a single EC number often encompasses multiple KOs corresponding to distinct isozymes or paralogs catalyzing the same reaction, making it impossible to distinguish between them on the basis of EC alone — the fallback therefore returns all KOs associated with that EC as a set. Second, and more consequentially for pathway scoring, multi-subunit enzyme complexes may have each subunit assigned a separate KO but share the same EC number (e.g., the β-lysine 5,6-aminomutase α and β subunits, K01844 and K18011, both assigned EC 5.4.3.3). In such cases, the EC fallback cannot determine whether one or both subunits were detected; the scoring expression therefore uses OR logic for these pairs (e.g., K01844 OR K18011 for *KamD/E* in P4), reflecting the annotation ambiguity rather than the true biochemical requirement. This is a pragmatic compromise widely adopted in metagenomics-based pathway inference, particularly for fragmented environmental assemblies where co-detection of both subunits in the same MAG is not always possible even when both are encoded [5]. The impact on pathway completeness scores is expected to be conservative: OR logic treats detection of either subunit as satisfying that pathway step, which may slightly inflate scores for MAGs encoding only one subunit. However, pathway completeness thresholds were intentionally calibrated conservatively to minimize the influence of such fallback-based inflation.

**Within-community functional redundancy (FRIa):** For each sample *j* and function *k*, all MAGs encoding the corresponding metabolic module were identified, and their contributions were weighted by relative MAG abundance. Contributions were normalized to probability weights (*p*ᵢ,ⱼ,ₖ), and Shannon entropy was calculated as Hⱼ,ₖ = −∑ᵢ *p*ᵢ,ⱼ,ₖ log(*p*ᵢ,ⱼ,ₖ). To account for differences in the number of functional providers, entropy was normalized by the logarithm of provider richness (*S*ⱼ,ₖ), yielding the within-community functional redundancy index: FRIaⱼ,ₖ = Hⱼ,ₖ / log(*S*ⱼ,ₖ). FRIa ranges from 0, indicating dominance by a single provider, to 1, indicating evenly distributed contributions among multiple taxa.

**Between-community functional redundancy (FRIb):** To quantify convergence or divergence in functional provider identity across communities exposed to identical environmental conditions, a between-community redundancy index was computed. For each function within a given environment, abundance-weighted provider vectors were compared pairwise across replicate samples using Bray-Curtis dissimilarity, and similarity was defined as FRIb = 1 − Bray-Curtis. High FRIb values indicate convergent recruitment of the same dominant functional taxa across communities, whereas low values indicate that equivalent functions are implemented by distinct taxa. FRIb values were summarized across all pairwise comparisons for each function and environment.

**Linking assembly-process fractions to physicochemical stress proxies:** To assess whether the magnitude of deterministic assembly varied along physicochemical gradients, we related Stegen-derived process fractions to a panel of stress proxies measured at week 8. The stress variables considered included total short-chain carboxylates (SCCAs), undissociated SCCAs, and H₂ partial pressure. These continuous variables were discretized into stress bins following two constraints adapted from published guidelines: (1) bins had to be statistically separable in their stress values (pairwise Wilcoxon tests across bins with Holm correction; α = 0.05); and (2) each bin had to retain sufficient sample size (minimum n = 6 observations per bin). We attempted up to K = 7 bins per stress variable using approximately equal-count (quantile) binning, and iteratively merged adjacent bins until both constraints were met; if constraints could not be satisfied, binning was not applied for that variable/pairing scheme. Associations between process fractions and stress proxies were evaluated at week 8 using linear models of the form: *fraction_process* ~ *stress*, fitted separately by Domain (Bacteria/Archaea).

At week 8, the fraction of selection-driven assembly covaried with short-chain carboxylate stress metrics. In bacterial communities, homogeneous selection increased with undissociated SCCA levels (*R*^2^ = 0.795, P = 0.00699), and heterogeneous selection increased with total SCCA concentration (*R*^2^ = 0.720, P = 0.0159). In archaeal communities, heterogeneous selection showed a strong positive association with undissociated SCCAs (*R*^2^ = 0.979, P = 2.29 × 10^-5^).

**Effect of yeast extract supplementation on gene-level functional profiles.** To assess whether yeast extract (YE) supplementation, included to support auxotrophic community members, introduced systematic shifts in the functional gene composition of the enriched communities, we performed a targeted MaAsLin2 analysis contrasting +YE versus -YE conditions at pH 6.5 in glucose-amended microcosms, using inoculum as a random effect. Full results are provided in Supplementary Table S18. YE supplementation was associated with significant enrichment or depletion of 1,971 KOs (FDR-corrected *q* < 0.05). The large majority of significant associations (80%) reflected lower relative abundance under +YE conditions, consistent with a broad reduction in biosynthetic gene expression when exogenous amino acids and cofactors are provided. The most strongly and reproducibly suppressed KOs included cysteine uptake and processing functions (cysteine transport protein K26604, O-acetylserine/cysteine efflux transporter K15268, L-cysteine desulfidase K26607; *Q* < 10^-12^), consistent with reduced demand for cysteine acquisition when it is already supplied by the yeast extract. Succinate dehydrogenase membrane subunits, glyoxylate cycle enzymes (malate synthase, isocitrate lyase), and hydrogenase maturation factors (*hypC, hypD, hypF*) were also significantly depleted under +YE conditions, suggesting reduced investment in anaerobic respiratory chains and Ni-Fe hydrogenase assembly when exogenous nutrients relax metabolic constraints. Conversely, YE supplementation was associated with enrichment of sporulation-related genes (29 KOs across sporulation stages IV and V, including stage V sporulation proteins K and AF, versus one depleted; mean effect +4.6), ribosome assembly GTPases (K06948, K14540), and amino acid import functions (aspartate/glutamate transport system; K10039/K10040). The coordinated enrichment of late-stage sporulation markers under +YE conditions suggests that nutrient availability may trigger dormancy programs in a subset of community members once active fermentation substrates are depleted. Flagellar assembly and chemotaxis genes were predominantly suppressed under +YE conditions, with the exception of a small subset of CheC/CheD-type chemotaxis adaptation proteins. Collectively, these results indicate that YE supplementation reshapes the functional gene profile of the community primarily by relieving biosynthetic burden and modulating stress-associated programs, without substantially altering the terminal electron-sink pathway organization that constitutes the main subject of this study.

## **Supplementary Figures**

**Figure S1. Separation of controls from biological samples.** *Dissimilarities were computed using the Bray-Curtis index.* **A,** Dissimilarity to the control centroid in the full PCoA space. **B,** Minimum dissimilarity to the nearest control. Controls consisted of a ZymoBIOMICS Gut Microbiome Standard (positive control) and a negative extraction control to detect reagent- or kit-derived contaminants.


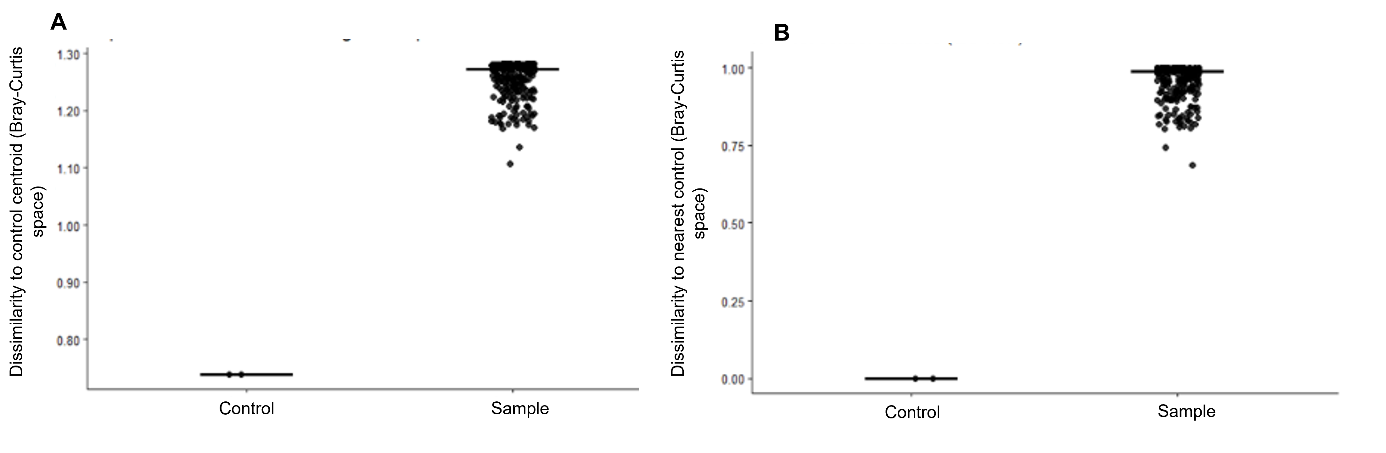


**Figure S2. Time-resolved changes in microbial diversity under controlled anaerobic digestion enrichments. A**, Shannon index computed for bacterial and archaeal communities declines over sequential transfers in glucose- and cellobiose-fed microcosms. **B,** Diversity trajectories for all substrate treatments*.*

**Figure S3:** **Temporal change in the fraction of communities assembled by environmental selection *vs.* stochastic processes at different taxonomic levels.**


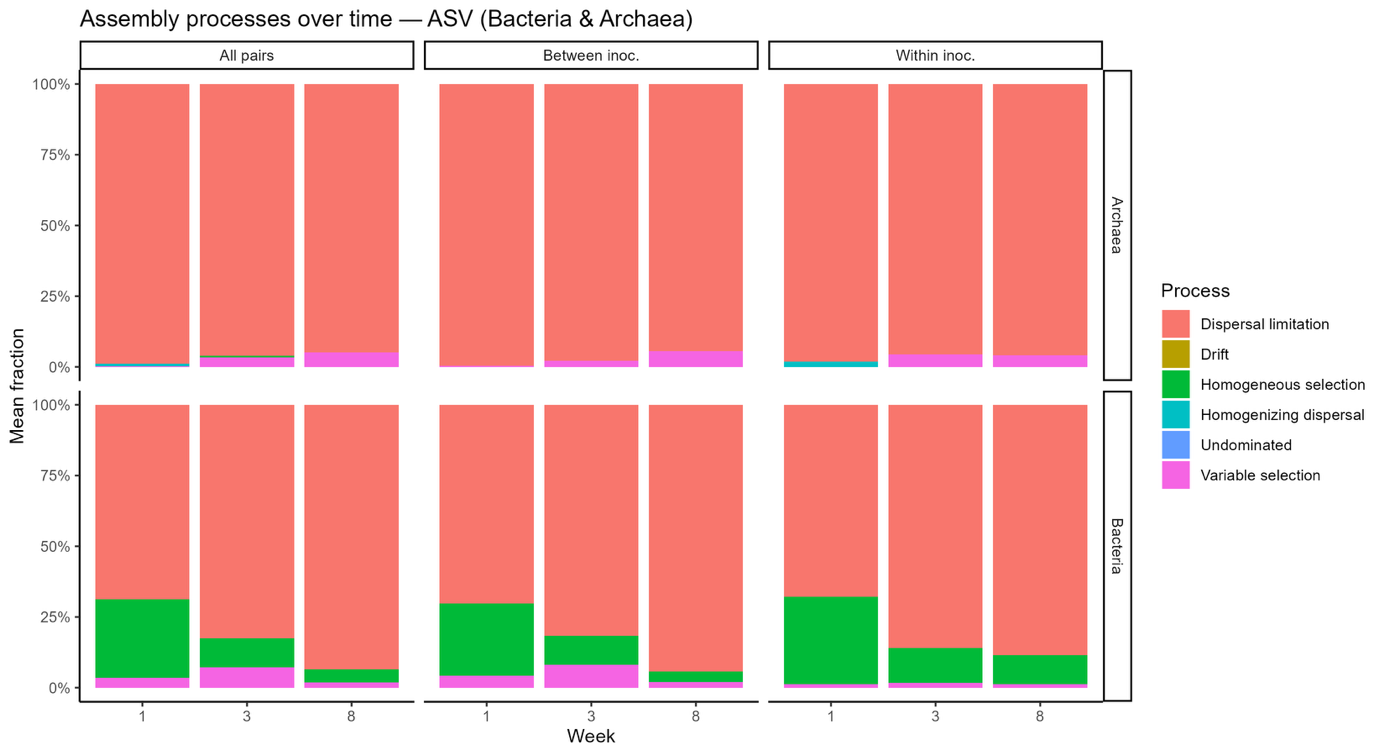


 **Figure S4:** **Bacterial community structure at week 8 across inoculum at the genus level.**

**Figure S5:** **Bacterial community structure at week 8 across inoculum at the family level.**

**Figure S6:** **Bacterial community structure at week 8 across inoculum at the order level.**

 **Figure S7: Temporal dynamics of bacterial community composition across successive transfers for communities enriched on cellobiose, at pH=5.5 and seeded from Inoculum 1.**


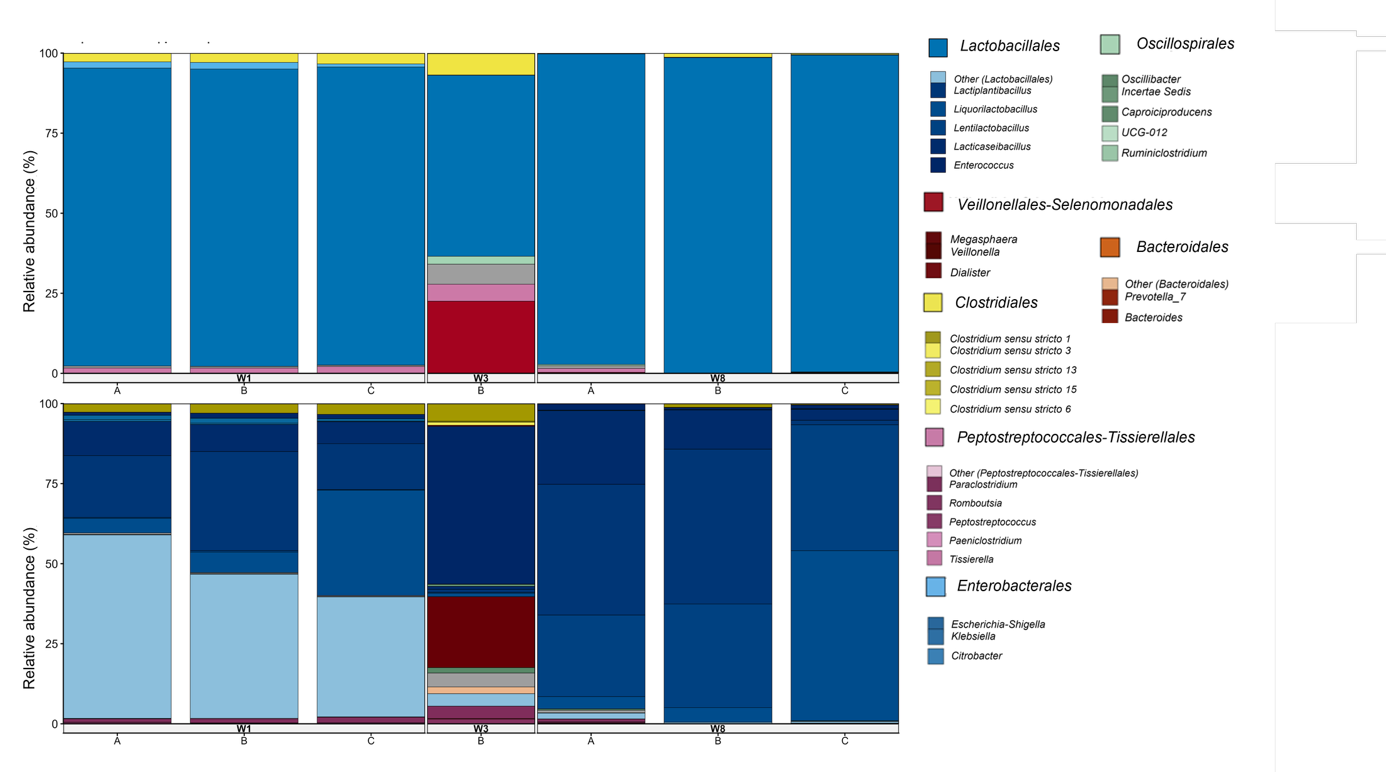


**Figure S8: Temporal dynamics of bacterial community composition across successive transfers for communities enriched on cellobiose, at pH=5.5 and seeded from Inoculum 2.**


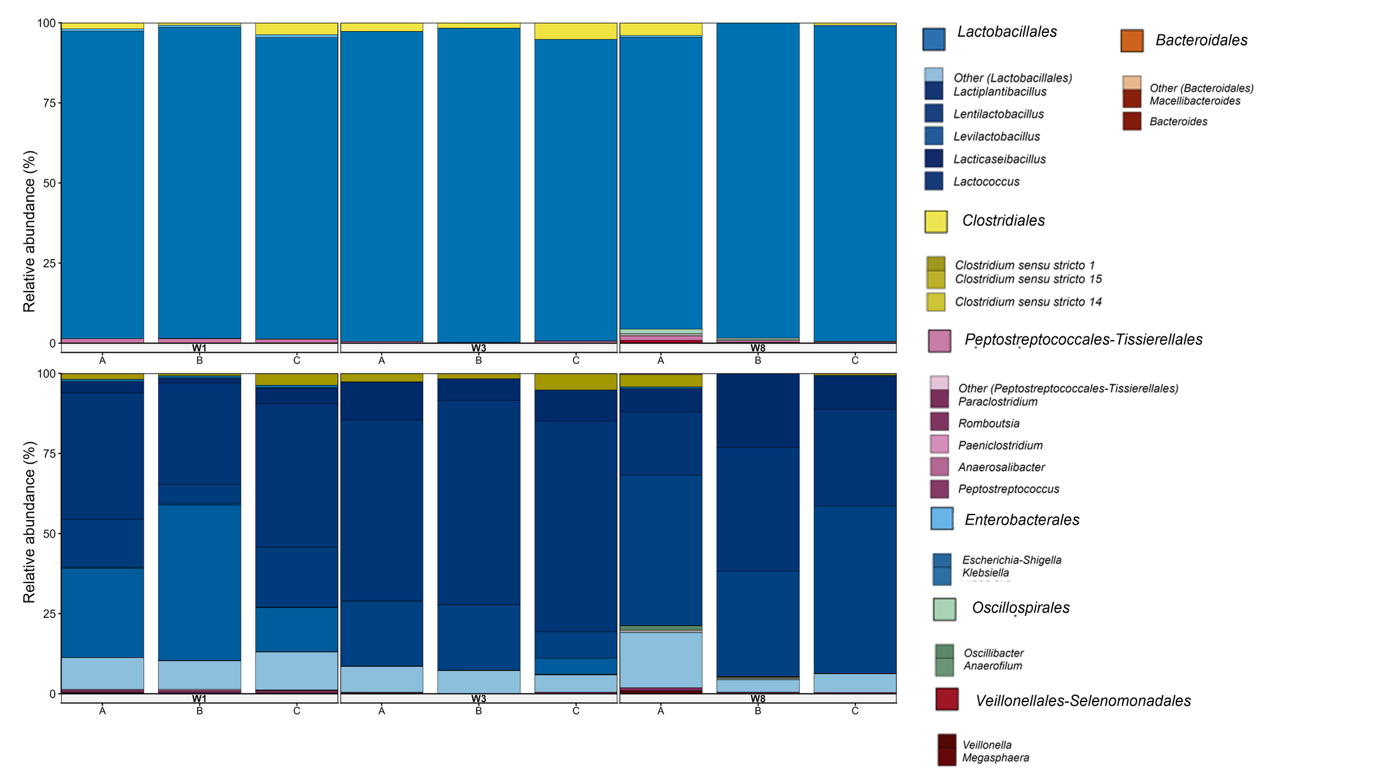


 **Figure S9: Temporal dynamics of bacterial community composition across successive transfers for communities enriched on cellobiose, at pH=5.5 and seeded from Inoculum 3.**


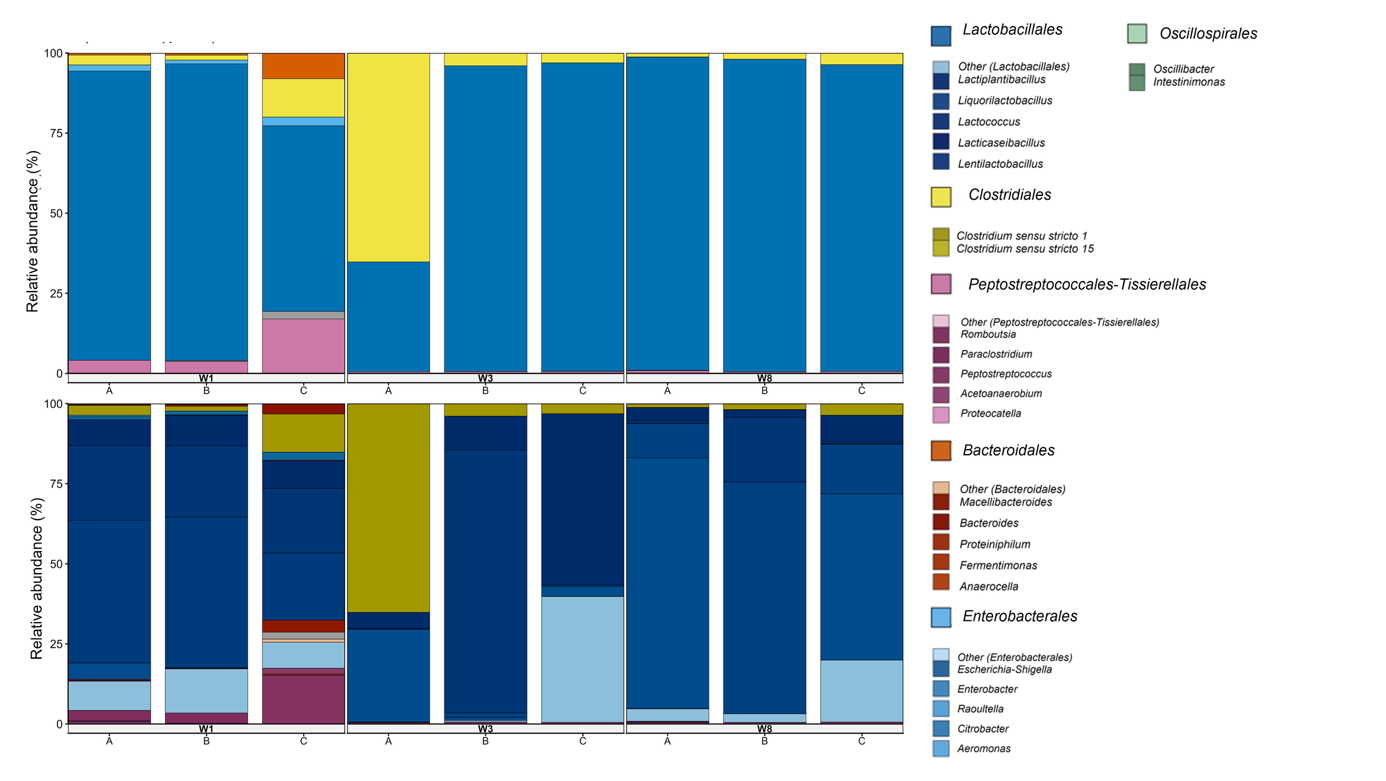


**Figure S10: Temporal dynamics of bacterial community composition across successive transfers for communities enriched on cellobiose, at pH=6.5 and seeded from Inoculum 3.**


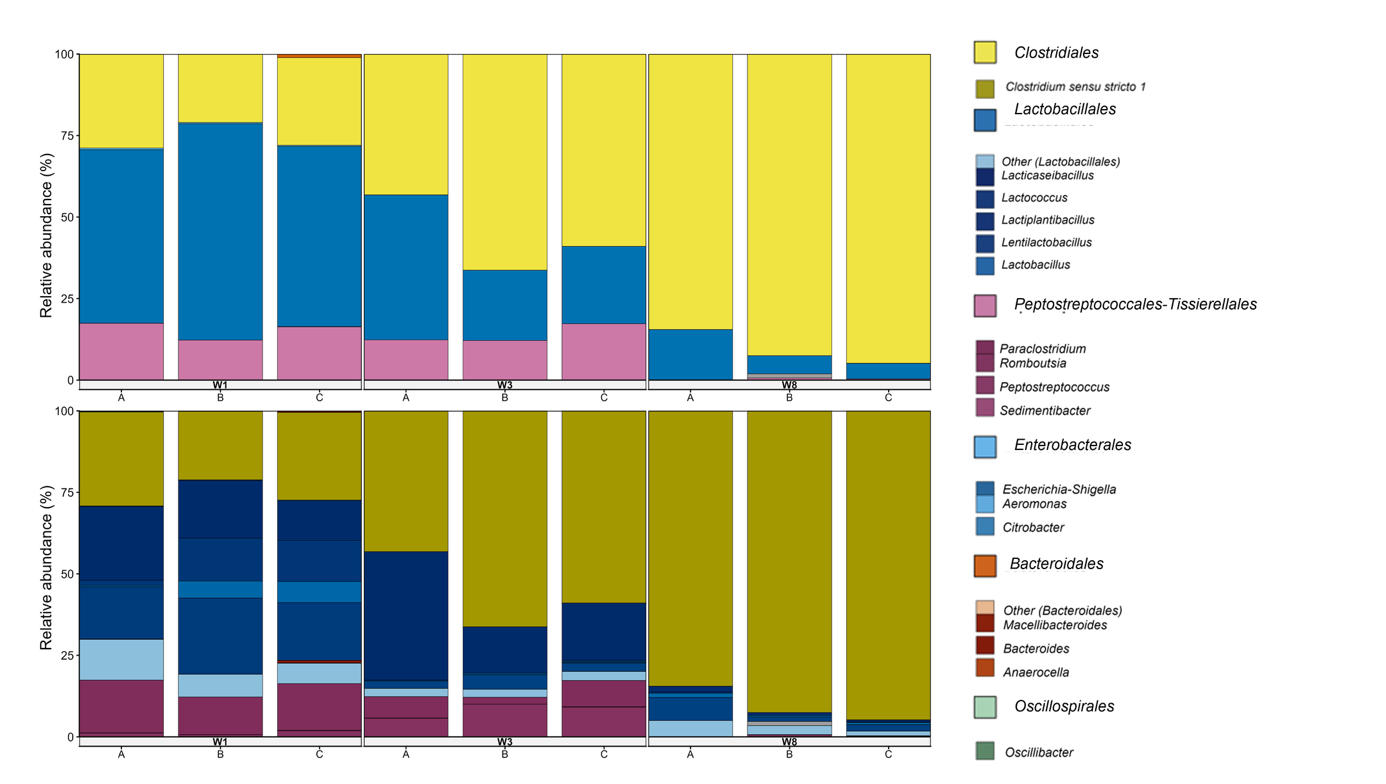


**Figure S11: Temporal dynamics of bacterial community composition across successive transfers for communities enriched on cellobiose, at pH=7.5 and seeded from Inoculum 2.**


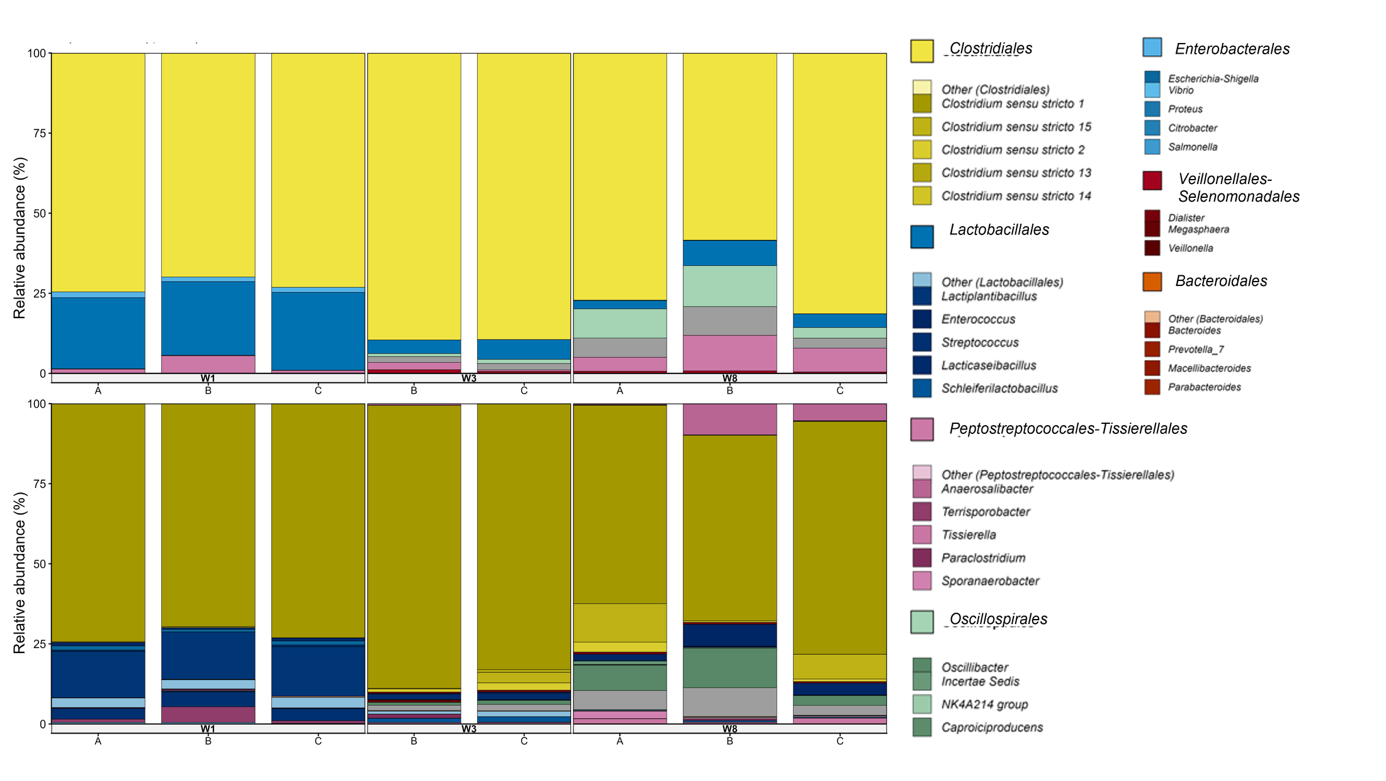


**Figure S12: Temporal dynamics of bacterial community composition across successive transfers for communities enriched on glucose, at pH=5.5 and seeded from Inoculum 3.**


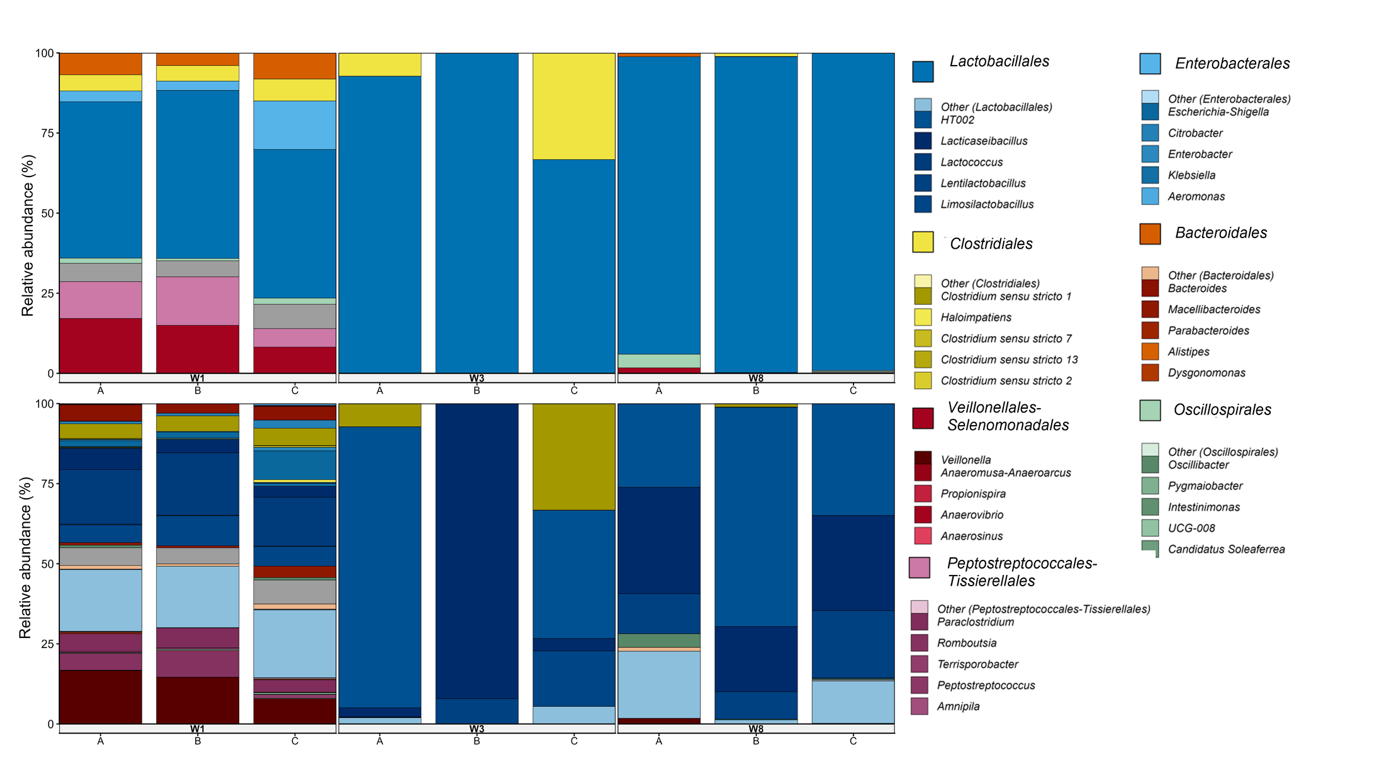

 **Figure S13: Temporal dynamics of bacterial community composition across successive transfers for communities enriched on glucose, at pH=7.5 and seeded from Inoculum 2.**


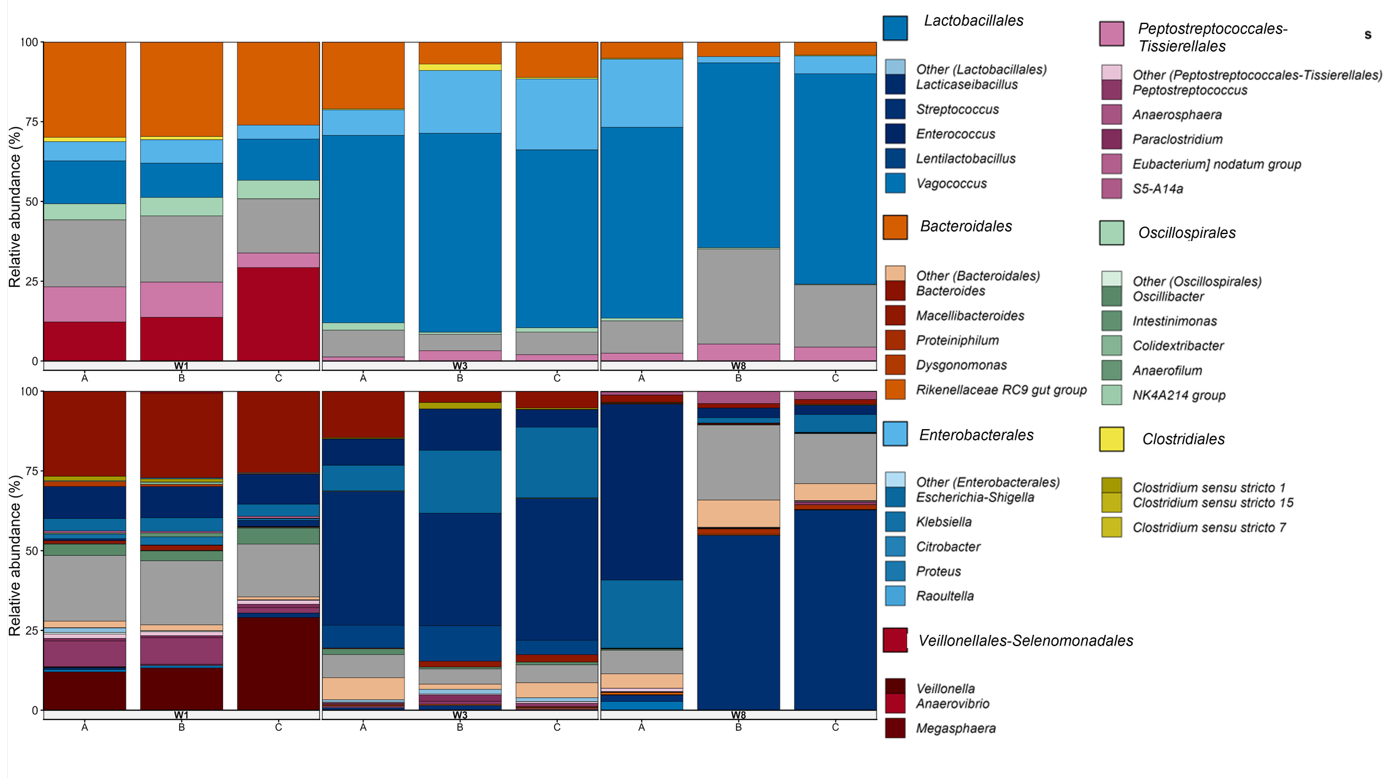


**Figure S14:** **Temporal dynamics of bacterial community composition across successive transfers for communities enriched on Glucose at pH 7.5.**

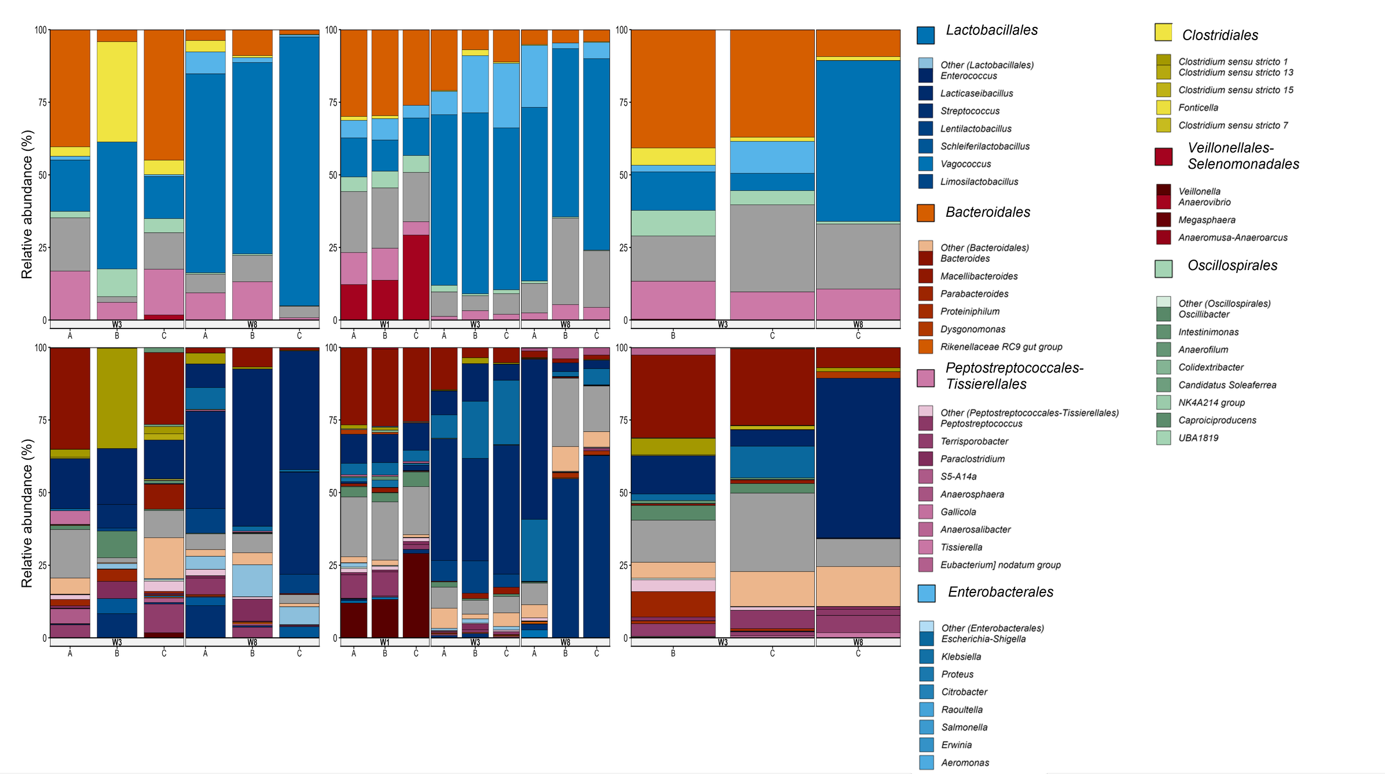


 **Figure S15: Temporal dynamics of bacterial community composition across successive transfers for communities enriched on cellobiose at pH 5.5.**

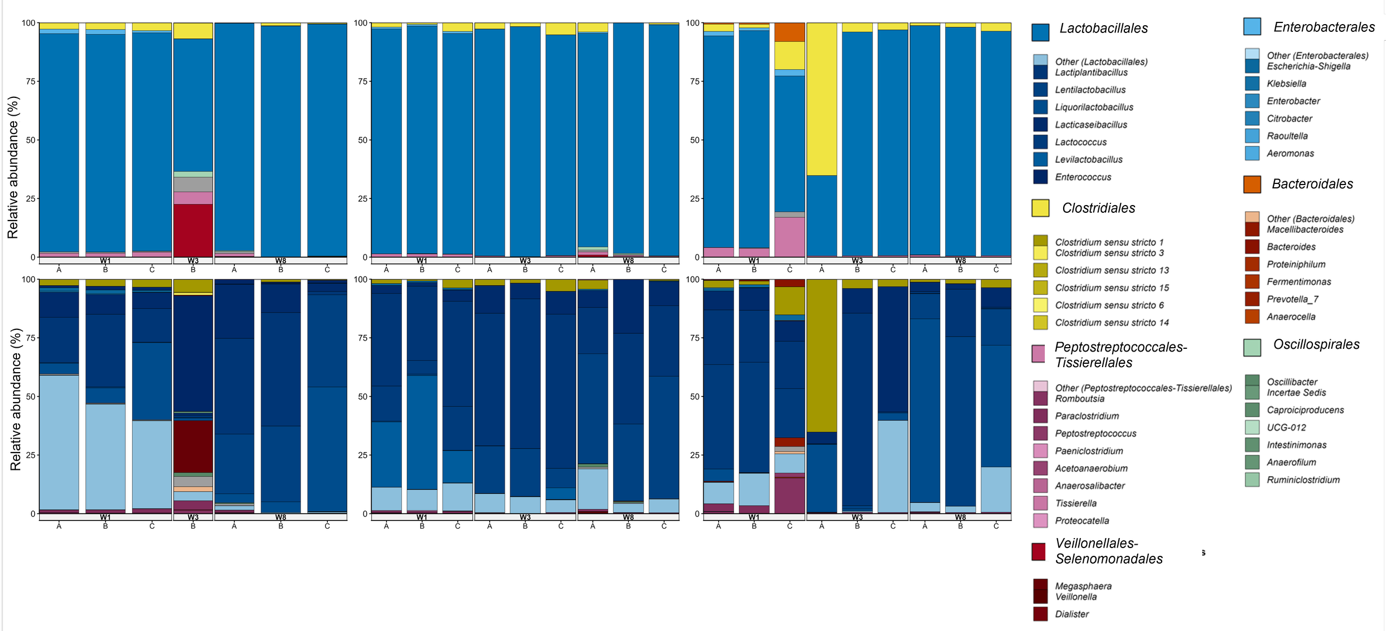


**Figure S16: Temporal dynamics of bacterial community composition across successive transfers for communities enriched on cellobiose at pH 6.5.**


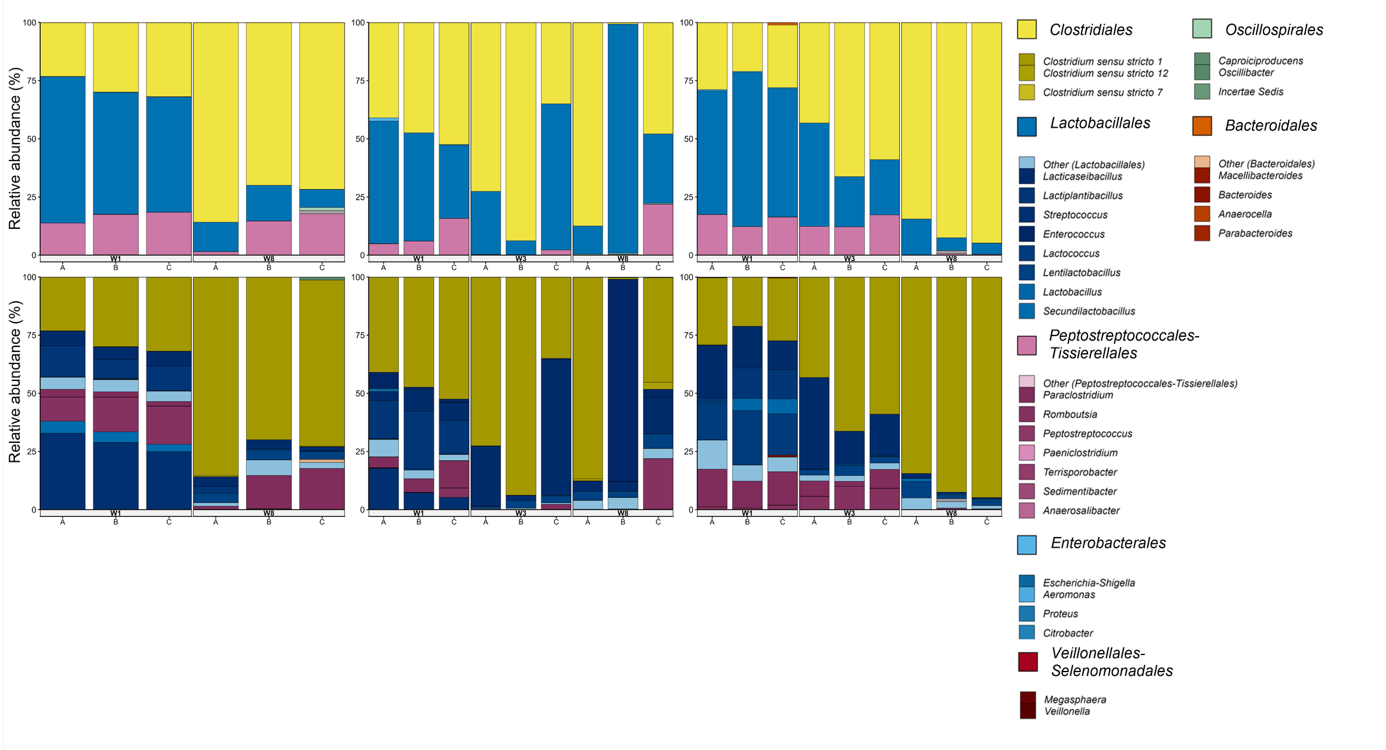


 **Figure S17: Shannon diversity across metabolic guilds and environmental conditions.**


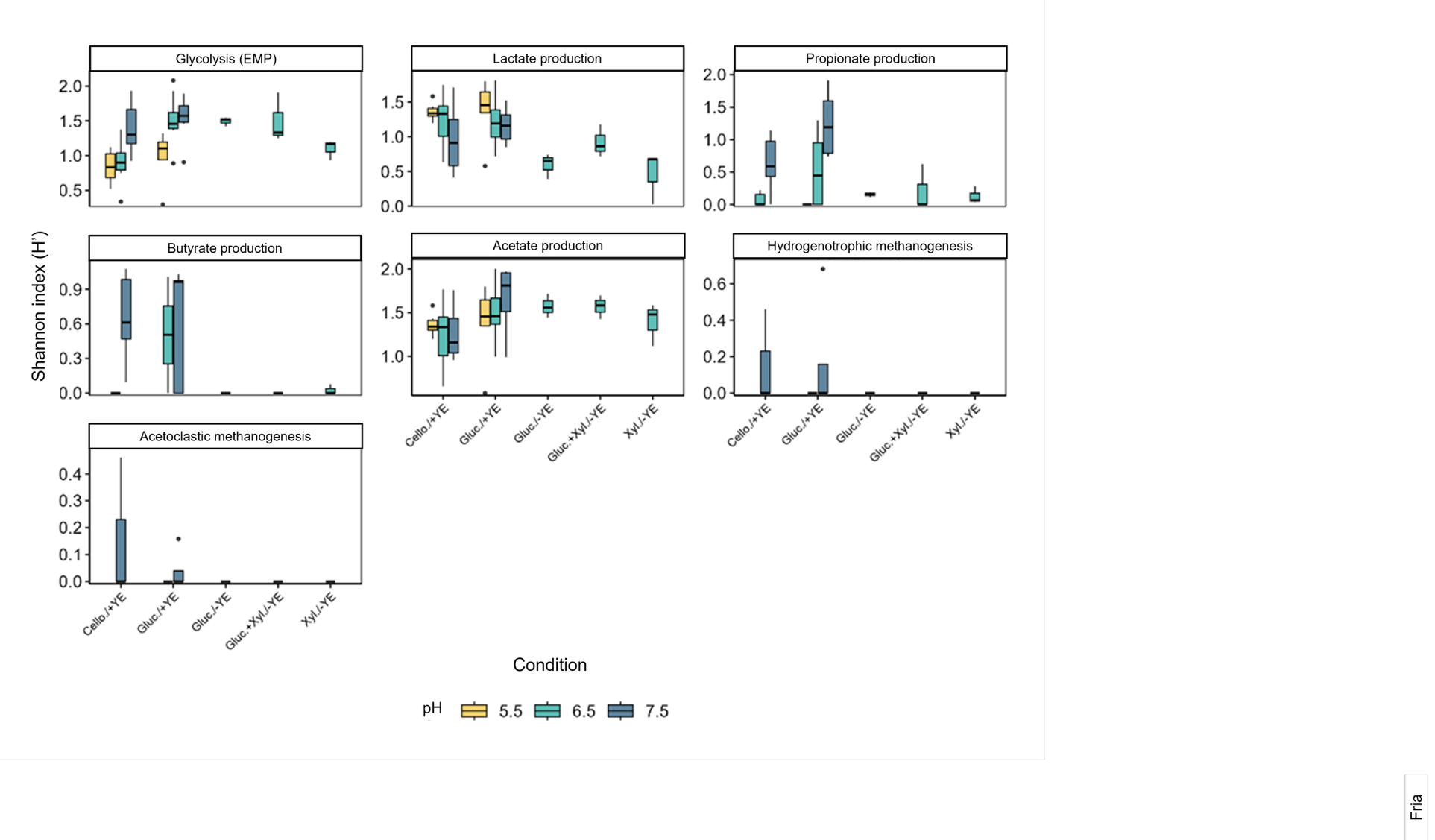


**Figure S18: Route allocation for propionate production across conditions, weighted by a genomic investment score defined as the mean gene copy number per pathway step (∑ KO copies / number of KOs in route), normalized to 100% across all three propionate routes (P1-P3) per MAG.**

 **Figure S19: Route allocation for butyrate production across conditions and completeness thresholds, weighted by a genomic investment score defined as the mean gene copy number per pathway step (∑ KO copies / number of KOs in route), normalized to 100% across all four butyrate routes (P1-P4) per MAG.**

**
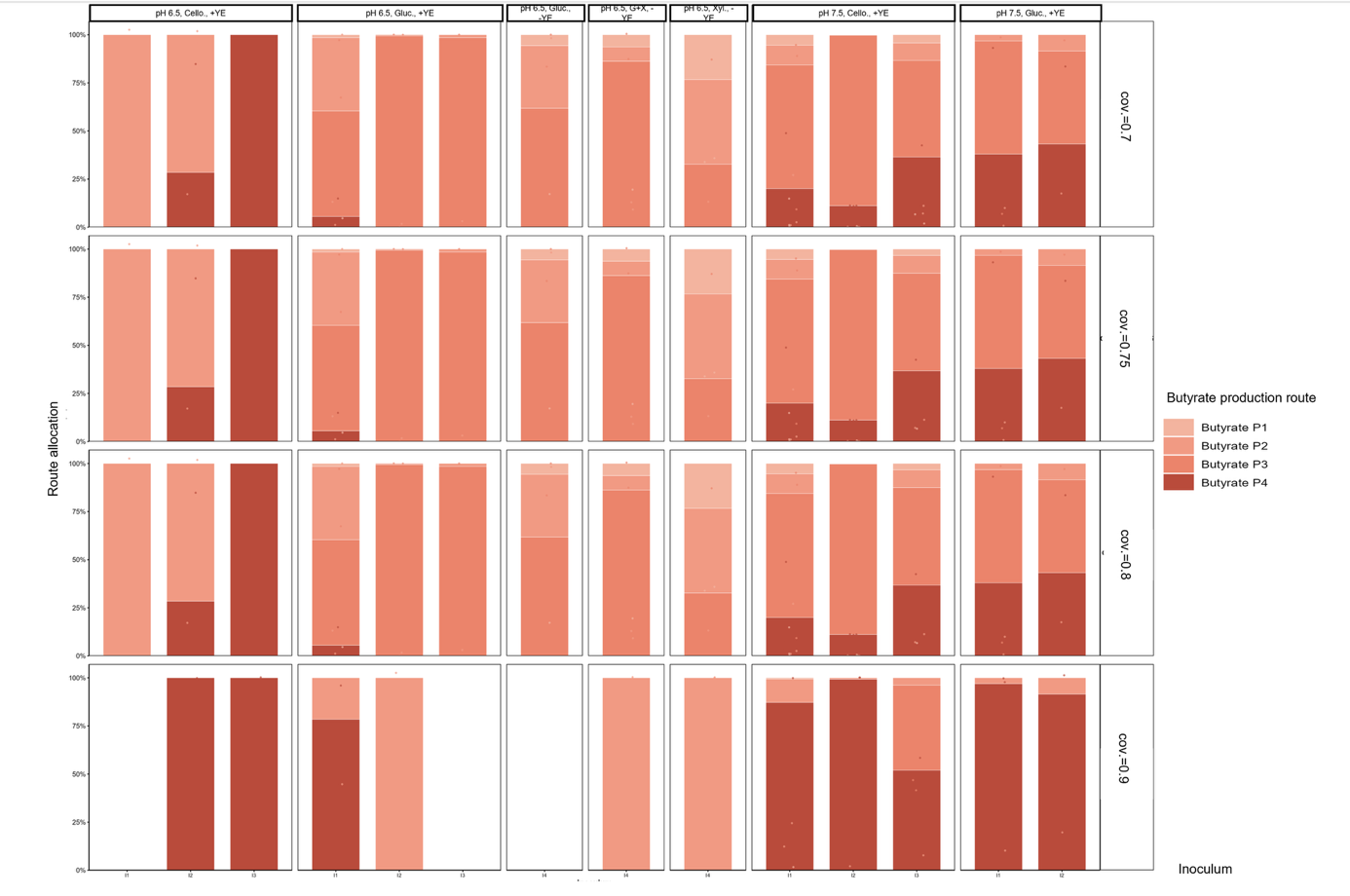
**

| **Supplementary Table S1. Relative abundance of metagenome-assembled genomes (MAGs) across samples.** |
| --- |
| **Supplementary Table S2. Sample metadata including inoculum origin, substrate treatment, pH condition, and experimental replicate information for metagenomic analyses.** |
| **Supplementary Table S3. MAG quality metrics and completeness/contamination statistics.** |
| **Supplementary Table S4. Taxonomic assignment of MAGs based on GTDB-Tk.** |
| **Supplementary Table S5. Curated anaerobic digestion pathway definitions and KO-based classification rules.** |
| **Supplementary Table S6. Gene-level differential abundance results across environmental contrasts.** |
| **Supplementary Table S7. Statistical tests for  within-community functional redundancy (FRIa): pairwise Dunn tests with BH correction (upper block), overall Kruskal-Wallis tests across all environments (middle block), and Kruskal-Wallis tests stratified by substrate (lower block).** |
| **Supplementary Table S8. Statistical tests for between-community functional convergence (FRIb).** |
| **Supplementary Table S9. Number of provider MAGs across metabolic functions and cultivation conditions.** |
| **Supplementary Table S10. Genomic investment scores across metabolic pathways and MAGs.** |
| **Supplementary Table S11. Number of provider MAGs across metabolic functions and completeness thresholds.** |
| **Supplementary Table S12. Occurrence of provider MAGs across metabolic functions and samples.** |
| **Supplementary Table S13. Provider MAG richness across cultivation conditions.** |
| **Supplementary Table S14. Raw metabolite measurements at the end of the enrichment.** |
| **Supplementary Table S15. Raw metabolite measurements of short-chain carboxylates and gaseous metabolites across transfers for glucose-, xylose-, and mixed-sugar enrichments.** |
| **Supplementary Table S16. Provider MAG assignments for individual metabolic functions.** |
| **Supplementary Table S17. Functional marker completeness and annotation source across provider MAGs.** |
| **Supplementary Table S18. Gene-level differential abundance results for glucose-enriched communities at pH 6.5.** |
| **Supplementary Table S19. Maximum relative abundance of butyrate providers assigned to multiple production pathways above completeness thresholds.** |

**References:**

1. Qiu S, Zhang X, Xia W. et al. Effect of extreme pH conditions on methanogenesis: Methanogen metabolism and community structure. Science of The Total Environment 2023;877:162702. https://doi.org/10.1016/j.scitotenv.2023.162702

2. Sun M, Liu B, Yanagawa K. et al. Effects of low pH conditions on decay of methanogenic biomass. Water Research 2020;179:115883. https://doi.org/10.1016/j.watres.2020.115883

3. Stegen JC, Lin X, Fredrickson JK, et al. Quantifying community assembly processes and identifying features that impose them. ISME J 2013;7:2069–2079. https://doi.org/10.1038/ismej.2013.93

4. Vila JCC, Liu Y-Y, Sanchez A. Dissimilarity–Overlap analysis of replicate enrichment communities. ISME J 2020;14:2505–2513. https://doi.org/10.1038/s41396-020-0702-7

5. Vital M, Howe AC, Tiedje JM. Revealing the Bacterial Butyrate Synthesis Pathways by Analyzing (Meta)genomic Data. mBio 2014;5:10.1128/mbio.00889-14. https://doi.org/10.1128/mbio.00889-14

6. Christensen R, Wang YHD, Arnoldini M, et al. Abundance-weighted pathway mapping demonstrates family-level structure of butyrate and propionate production across the human gut microbiome. ISME communications. 2026 Jan;6(1):ycag075. https://doi.org/10.1093/ismeco/ycag075
